# The relationship between remotely-sensed spectral heterogeneity and bird diversity is modulated by landscape type

**DOI:** 10.1016/j.jag.2024.103763

**Published:** 2024-04

**Authors:** Dominika Prajzlerová, Vojtěch Barták, Petr Keil, Vítězslav Moudrý, Markéta Zikmundová, Petr Balej, François Leroy, Duccio Rocchini, Michela Perrone, Marco Malavasi, Petra Šímová

**Affiliations:** a Department of Spatial Sciences, Faculty of Environmental Sciences, Czech University of Life Sciences Prague, Kamýcká 129, 165 00 Praha – Suchdol, Czech Republic; b Department of Mathematics, Informatics amd Cybernetics, Faculty of Chemical Engineering, University of Chemistry and Technology, Prague, Technická 5, Praha 6, 16628, Prague, Czech Republic; c BIOME Lab, Department of Biological, Geological and Environmental Sciences, Alma Mater Studiorum University of Bologna, via Irnerio 42, 40126, Bologna, Italy; d Department of Chemistry, Physics, Mathematics and Natural Sciences, University of Sassari, Via Vienna 2, 07100 Sassari, Italy

**Keywords:** Bird species richness, Habitat modeling, Landsat 8, Remote sensing, Spectral heterogeneity

## Abstract

To identify areas of high biodiversity and prioritize conservation efforts, it is crucial to understand the drivers of species richness patterns and their scale dependence. While classified land cover products are commonly used to explain bird species richness, recent studies suggest that unclassified remote-sensed images can provide equally good or better results. In our study, we aimed to investigate whether unclassified multispectral data from Landsat 8 can replace image classification for bird diversity modeling. Moreover, we also tested the Spectral Variability Hypothesis.

Using the Atlas of Breeding Birds in the Czech Republic 2014-2017, we modeled species richness at two spatial resolutions of approx. 131 km^2^ (large squares) and 8 km^2^ (small squares). As predictors of the richness, we assessed 1) classified land cover data (Corine Land Cover 2018 database), 2) spectral heterogeneity (computed in three ways) and landscape composition derived from unclassified remote-sensed reflectance and vegetation indices. Furthermore, we integrated information about the landscape types (expressed by the most prevalent land cover class) into models based on unclassified remote-sensed data to investigate whether the landscape type plays a role in explaining bird species richness.

We found that unclassified remote-sensed data, particularly spectral heterogeneity metrics, were better predictors of bird species richness than classified land cover data. The best results were achieved by models that included interactions between the unclassified data and landscape types, indicating that relationships between bird diversity and spectral heterogeneity vary across landscape types.

Our findings demonstrate that spectral heterogeneity derived from unclassified multispectral data is effective for assessing bird diversity across the Czech Republic. When explaining bird species richness, it is important to account for the type of landscape and carefully consider the significance of the chosen spatial scale.

## Highlights

• Spectral heterogeneity performs better than land cover data to explain bird richness.
• Interactions with landscape types improve predictive power of spectral heterogeneity.
• The predictive power of spectral heterogeneity is influenced by scale.

## Graphical abstract

**Figure:**
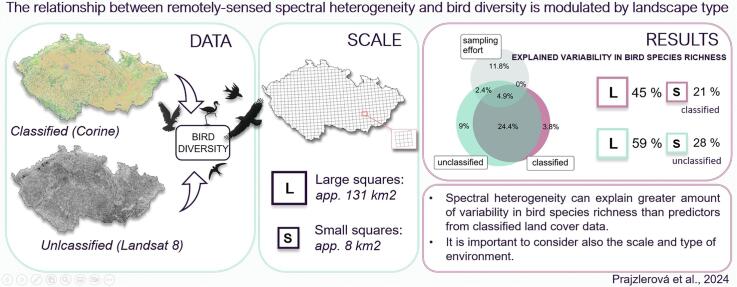

## 1 Introduction

Explaining why biodiversity varies in space, and being able to accurately map it, are crucial areas of research in basic ecology, with implications for applied nature and landscape protection. However, it is logistically impossible to conduct sampling of species distributions that would be comprehensive enough to determine biodiversity over large regions, and fast enough to assess changes in biodiversity over time. Having a set of simple indicators (proxies) of biodiversity would be beneficial to researchers and conservationists. For instance, in the context of bird diversity, specific bird species occurrences (Morelli et al., 2017, Roth and Weber, 2008) and/or landscape composition and structure (Billeter et al., 2008, Cooper et al., 2020, Lausch et al., 2016, Morelli et al., 2018, Moudrý et al., 2023b, Panda et al., 2021) have been proposed as useful indicators of species/functional diversity. Moreover, it is well-established that high habitat heterogeneity is linked to high biodiversity (Stein et al. 2014).

With the increasing availability of open satellite remote-sensed data, landscape composition, structure, and heterogeneity can be described in various ways. A traditional method is based on the supervised classification of multispectral imagery (Ma et al., 2017, Tang et al., 2007 Zhang and Zhu, 2011), i.e., distinguishing individual vegetation types or land cover classes, such as pastures, forests, deserts, or wetlands (He et al., 2015, Leyequien et al., 2007), and deriving landscape characteristics from these classified maps. As shown in many studies (Lindenmayer et al., 2002, Morelli et al., 2018, Walz, 2011), even simple landscape metrics such as the area of individual classes, patch richness, or edge density (Šímová and Gdulová, 2012) can indicate taxonomic and/or functional diversity of animals, albeit with some limitations.

An important limitation in species diversity modeling based on classified land cover information data lies in the fact that the modeled link between biodiversity and landscape characteristics depends on spatial scale (extent and minimum mapping unit or pixel size, also known as grain or resolution (Schindler et al., 2013)), time and thematic resolution, and classification accuracy (Šímová et al. 2019). Several freely available products provide *classified* land cover data at the extent of whole countries or continents (e. g. CLC – Corine Land Cover, NLCD – National Land Cover Database) or even worldwide (e. g. GLC – Global Land Cover Project). The spatial resolution of such classified land cover maps is usually coarse (for example, the minimum mapping unit of Corine is 0.25 km^2^, and the pixel size of GLC is 1 km^2^). It means that small (but sufficiently large for a species) habitat patches and transitions between the classes (i.e. ecotones providing a specific and valuable type of habitat) are overlooked in these datasets (Šímová et al., 2019, St-Louis et al., 2014). The thematic resolution, i.e., the character and number of classes distinguished in the classified map, represents another possible limitation. The definition of classes is typically guided by human perception of landscape, which may not reflect the true habitat requirements of species (Coops and Wulder, 2019, Gillespie et al., 2008, Zitske et al., 2011). For example, bird habitats are, among other factors, influenced by vegetation structure (Basile et al., 2021), which is not captured in the classified data - the overall configuration and the relative proportions of vegetation in the understorey, shrub layer, and canopy are crucial determinants affecting aspects such as nest predation, the quantity and availability of food resources, and microclimatic conditions. Consequently, these factors significantly contribute to the quality of nesting habitat and nest success (Bradley and Fleishman, 2008). In addition, the land cover classification accuracy is often low, around 65–80% (Chen et al., 2015, Gómez et al., 2016, Gong et al., Jun 2013). Thus, up to a third of all pixels in the land cover data can be misclassified. This inherently introduces errors into the models explaining and predicting biodiversity. Last but not least, due to the time-consuming classification process (which adds to its expense), classified remote sensing-based maps are typically available only for several time points, which limits analyses of landscape (and biodiversity) changes over time. Because of all these drawbacks, the reliability of biodiversity indicators derived from classified land cover maps can be questionable.

Fortunately, there is an alternative to the use of classified land cover data: we can derive predictors of species distributions and diversity from *unclassified* multispectral remote-sensed data (Culbert et al., 2012, Duro et al., 2014, Farwell et al., 2020, Hunt et al., 2022, Sheeren et al., 2014) that do not suffer from the abovementioned limitations (Pettorelli et al., 2014, Sheeren et al., 2014). These predictors, derived from reflectance values from individual bands or derived vegetation indices, offer insights into soil and vegetation moisture, biomass content, and vegetation greenness due to the unique spectral responses of different surfaces. Vegetation indices are used for assessing vegetation cover through simple and effective algorithms, utilizing observations in two or more spectral bands (Bannari et al., 1995, Xue and Su, 2017). These unclassified data preserve the original information in each pixel, avoiding distortions caused by the exclusion of local characteristic (Gottschalk et al., 2005), subjective classification (St-Louis et al., 2014), and/or misclassification (Shao and Wu, 2008). Unclassified data thus represent an inexpensive way of consistent and regular acquisition of information on landscape structure over large areas (Foody and Cutler, 2003, Muldavin et al., 2001, Rocchini et al., 2010). Therefore, there is a need for studies that compare the ability of unclassified multispectral satellite data and classified land cover maps to explain animal diversity (Culbert et al., 2012, Duro et al., 2014, Hunt et al., 2022 or Sheeren et al., 2014).

Both classified and unclassified data can be used for characterizing landscape heterogeneity for the purposes of modeling species diversity (Tuanmu and Jetz, 2015). Landscape heterogeneity is the main factor when mapping species diversity – areas with higher structural and compositional differences in vegetation and terrain can host more species due to the higher number of available niches (Hortal and Lobo, 2005; Stein et al., 2014). Analogously to the traditional heterogeneity metrics derived from classified land cover maps [e.g. Patch Richness and similar (Adler and Jedicke, 2022; Plexida et al., 2014, Schindler et al., 2015, Schindler et al., 2013)], spectral heterogeneity can be calculated directly from unclassified multispectral data. The association of such 'spectral heterogeneity' or 'spectral variability' with species diversity has been proposed in the *Spectral Variation Hypothesis* (SVH) (Palmer et al., 2000, Palmer et al., 2002), which suggests that environmental heterogeneity correlates with spectral heterogeneity of unclassified remote-sensed multispectral imagery.

SVH has been tested on several groups of organisms, including vascular plants (e.g., Foody and Cutler, 2003, Levin et al., 2007, Perrone et al., 2022, Rocchini et al., 2014, Rocchini et al., 2004, Rugani and Rocchini, 2017), mammals (Oeser et al., 2020), and birds (Bino et al., 2008, Farwell et al., 2021, Hunt et al., 2022, Sheeren et al., 2014, St-Louis et al., 2014, Wood et al., 2013). A higher number of studies on vascular plants is available due to a more direct mechanistic link between plant diversity and spectral heterogeneity (Ustin and Gamon, 2010): the multispectral remote-sensed images contain direct information about plants and trees on the Earth's surface, while where animals are concerned, the imagery describes only the spectral heterogeneity of their habitats.

SVH-based biodiversity modeling, i.e., the use of unclassified spectral data for biodiversity modeling, has both advantages and limitations. The relationship between species diversity and spectral heterogeneity can depend on the landscape type (i.e. type of environment) (Perrone et al., 2022, Schmidtlein and Fassnacht, 2017), and SVH is, therefore, not valid universally across all landscape types. While Perrone et al. (2023) included the most prevalent land cover type in the analysis to account for various landscape types, Schmidtlein and Fassnacht (2017) employed a moving window approach to derive statistical links between spectral variability and species richness through space and time. At the same time, the relationship between diversity and spectral heterogeneity is likely scale-dependent, because scale-dependence is a general feature of species-environment models (McGarigal et al., 2016, Moudrý et al., 2023a, Moudrý and Šímová, 2012). The plethora of indices of spectral heterogeneity (reviewed by Wang and Gamon, 2019) is another issue. Indices differ in their theoretical background, computational cost, and can thus vary in their ability to predict biodiversity. For example, parameterized Rao's Q is a commonly used index of landscape heterogeneity (Torresani et al., 2018, Rocchini et al., 2021a, Rocchini et al., 2021b, Rocchini et al., 2018); however, its calculation for large areas is computationally costly. We thus see an opportunity to use indices that are easy to calculate, such as the coefficient of variation or standard deviation.

Here, we test if there is a relationship between bird species diversity and predictors of landscape structure and heterogeneity derived from (i) a standard classified land cover map (Corine) and (ii) unclassified multispectral satellite data (Landsat 8) at two spatial grains (131 km^2^ and 8 km^2^) over the extent of the Czech Republic. In addition, we also test the influence of the original image resolution (pixel size) on that relationship. We aim to compare the ability of these two groups of predictors to explain species diversity at different spatial scales across various landscape types. We expect that the predictors based on unclassified multispectral data will explain the bird species richness better, than those based on classified Corine data, particularly at the finer spatial grain, due to the coarse spatial resolution (minimum mapping unit of 0.25 km^2^) of Corine for such small sampling unit. We also hypothesize that bird communities may vary in their response to environmental predictors in different types of landscapes due to their diverse ecological requirements.

## 2 Methods

### 2.1 Study area

The study area (*approximately* 69,000 km^2^) is in Central Europe, situated within the Czech Republic. It consists mainly of hills, plateaus, and lowlands surrounded by mountains along the borders. The altitude ranges between 115 and 1603 m above sea level. The landscape consists mainly of temperate forests, farmlands, inland waters, and urban areas. The climate is temperate, with a regular alternation of four seasons.

### 2.2 Species data

**Figure 1:**
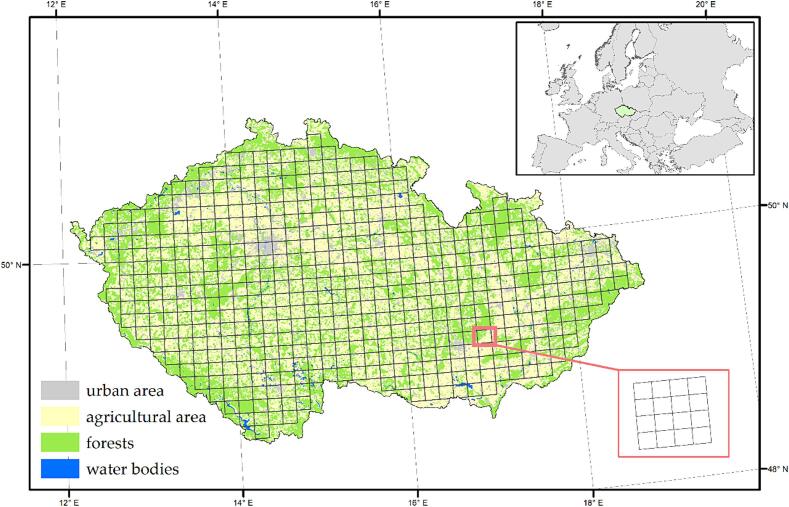
Czech Republic covered by the grid of the breeding bird Atlas: each large square (approx. 131 km^2^) is subdivided into 16 small squares (approx. 8 km^2^). The displayed land cover is obtained from the Corine database.

We used bird data from the Atlas of Breeding Birds in the Czech Republic 2014–2017 (Šťastný et al., 2021; hereinafter Atlas). The grid of the Atlas data consists of ‘large squares’ (10’ east longitude × 6’ north latitude; see Fig. 1), each of which is subdivided into 16 ‘small squares’ (Fig. 1). These grid sizes were used as the two grains of analysis evaluated in this study.

The presence and absence of each bird species were mapped specifically for the Atlas by hundreds of ornithologists (i) in all large squares and (ii) in randomly selected small squares (Fig. 3). At least one person undertook bird mapping in every large square; most of them were, however, mapped by several people. The ornithologists systematically and repeatedly searched for evidence of the breeding occurrence of each species in all habitats present in the particular large square. Following the standards used in Europe (Hagemeijer and Blair, 1997), they recorded occurrences in three categories defining the probability of breeding as 'A – possible breeding', 'B – probable breeding', and 'C – confirmed breeding'; the highest category found for a given square is recorded in the Atlas. Such broad-scale mapping was complemented by mapping in the grain of the randomly selected small squares, where the so-called 'one-hour survey' was applied. In this type of survey, the volunteers recorded the first detection of each species, regardless of abundance, during six time slots lasting for 10 minutes each.

We used the number of bird species (i.e., species richness, hereafter richness) as the index of diversity and as the main response variable in our models. To assess the influence of scale on the relationship between spectral heterogeneity and richness, we used both sizes of squares, large and small. As the species data in grid squares intersecting the country's border can be inconsistent, we used only grid squares lying entirely in the Czech Republic. We used all breeding categories (A, B, C) for modeling (Moudrý et al., 2017).

**Figure 2:**
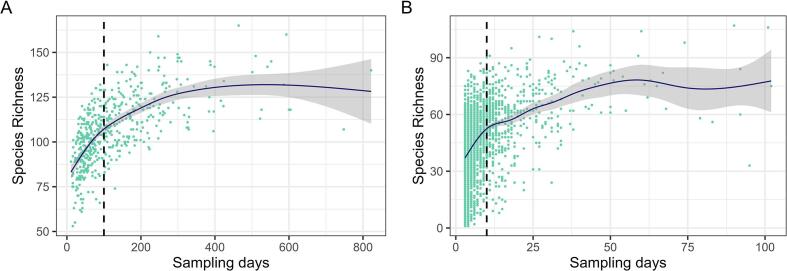
Relationship between species richness (number of species) and sampling effort (number of sampling days) in (A) large squares and (B) small squares. The Pearson correlation coefficients indicate a correlation of 0.60 (0.43 post-threshold) for large squares and 0.41 (0.35 post-threshold) for small squares. The blue line represents the trend estimated using the Loess smoothing method and the grey area represents its point wise 95% confidence bands. We considered in our analysis only the squares with more than (A) 100 or (B) 10 sampling days (black dashed lines) for large or small cells, respectively. The threshold was qualitatively selected by assessing the visual appearance of the scatterplot graphs.

**Figure 3:**
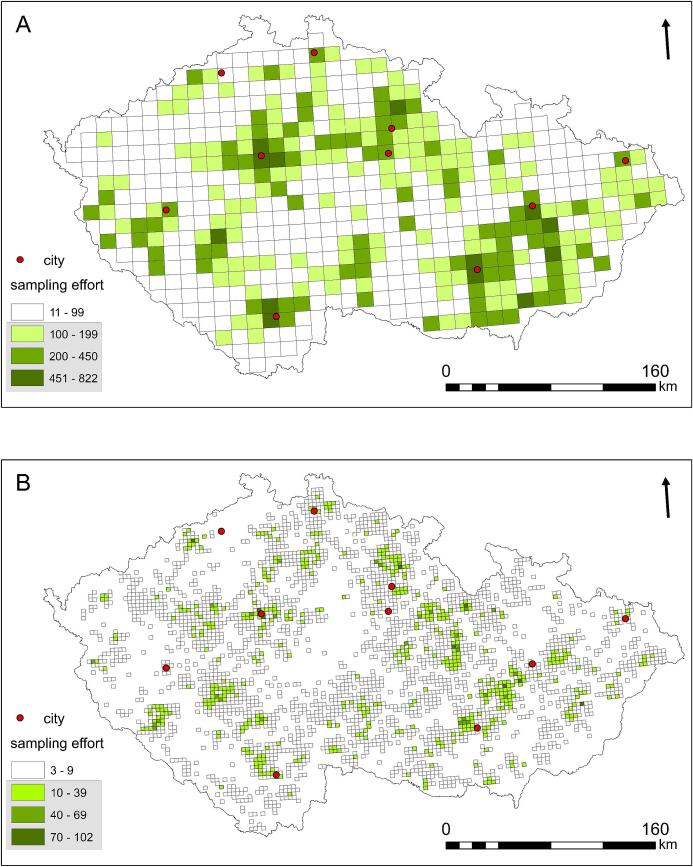
Spatial distribution of sampling effort. A: large squares, B: small squares. Colors indicate the number of sampling days. The dotted green squares represent those that were used in the models. The squares with the highest sampling effort match the locations of the largest cities within the Czech Republic. Empty spaces indicate areas that have not been mapped during preparation of the Bird Breeding Atlas of the Czech Republic

Although we originally had not expected significant sampling bias as the data are supposed to be systematically collected (see above and also Šťastný et al., 2021), we found a strong dependence between sampling effort and species richness (Fig. 2). Hence, we excluded the squares with fewer than 100 visits in the large squares (237 squares remaining) and those with fewer than 10 visits in the small squares (467 squares remaining) from our analysis (see Figure 2, Figure 3).

### 2.3 Classified predictors - land cover data

Land cover information was obtained from the Corine Land Cover (CLC2018) database, referring to the land cover/land use status in 2018. We distinguished seven classes (Table 1).

**Figure 4:**
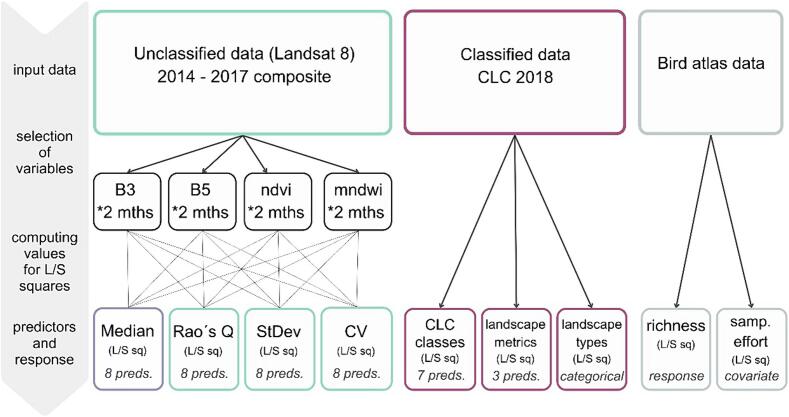
The schematic procedure of the acquisition of the variables utilized in our analyses. The diagram illustrates the process from raw input data through variable selection to the final predictors and response calculated for both small and large squares.

We used the classes described in Table 1 to calculate predictors of bird species richness (Fig. 4). Landscape composition was described by calculating the *area of the individual classes* within each square. To assess the landscape heterogeneity using land cover, we used three simple landscape metrics as recommended by Šímová et Gdulová (2012) due to their predictable behavior across spatial scales: (1) *Patch richness* (PR – number of unique classes of land cover in the sampling unit), (2) *Number of patches* (NP – number of CLC polygons in the sampling unit), and (3) *Largest patch area* (LP – the area of the largest polygon in the sampling unit).

**Table 1:** Corine classes used in our study and their description.

Corine class	Codes	Description	Abbreviation
Artificial surfaces	1	*Urban fabric; Industrial, commercial and transport units; Mine, dump and construction sites; Artificial, non-agricultural vegetated areas*	Urban
Coniferous forests	3.1.2	*Coniferous forests*	Coniferous
Broad-leaf and mixed forests	3.1.1; 3.1.3	*Mixed forest; Broad-leaved forest*	Leaves and mixed
Open vegetation	3.2, 3.3	*Scrub and/or herbaceous vegetationassociations; Open spaces with little or no vegetation*	Open vegetation
Arable land and permanent crops	2.1; 2.2	*Arable land; Permanent crops*	Agro
Pastures and heterogenous agricultural areas	2.3; 2.4	*Pastures; Land principally occupied by agriculture, withsignificant areas of natural vegetation; Annual crops associated with permanent crops; Complex cultivation patterns; Agro-forestry areas*	Natural agro
Wetlands and water bodies	4; 5	*Inland wetlands; Inland waters*	Water

### 2.4 Unclassified predictors - multispectral data

In addition to the classified Corine data, we used data from Landsat 8, the satellite imagery was downloaded from the Earth Engine Catalog (USGS Landsat 8 Level 2, Collection 2, Tier 1) with spatial resolutions of 30 and 100 m (with the aim of assessing the influence of the original image resolution on the resulting models), using rgee – an R package designed to interact with Google Earth Engine (Aybar et al., 2020). We restricted our analysis to image collections from 2014 to 2017, the same range as the Breeding Birds Monitoring data, calculated separately for 2 months, April and July. By April we wanted to capture the beginning of the vegetation season, assuming that spectral heterogeneity is highly variable during this annual period. To represent the peak of the vegetation season, characterized by full landscape coverage, we opted for July as a representative month.

To prepare individual bands for analysis, we first eliminated pixels of low quality (e.g. clouds, shadows, or other invalid data), we filtered the collection according to the properties “QA_PIXEL”, “QA_RADSAT” and “SR_QA_AEROSOL” in Google Earth Engine (Gorelick et al., 2017). Due to the presence of clouds and division into multiple acquisition paths, it was not feasible to utilize a single image from a specific date to cover the entire Czech Republic. We, therefore, used cloudless data from all years to create specific monthly composites for April and July. To address the issue of missing data in some pixels, we computed the percentage representation of valid pixels (those that were not removed during the collection filtering) for each square and removed squares containing less than 50% quality pixels, resulting in the removal of 11 large squares and 48 small squares (thus, a total of 226 large and 419 small squares remained, constituting the basis for all subsequent analyses in the study).

In addition to reflectances, vegetation indices were also employed as they were repeatedly shown to explain a significant portion of biodiversity (Hall et al., 2010; Levin et al., 2007, Oindo et al., 2000, Rocchini et al., 2014). Given the strong correlation observed among individual bands and vegetation indices, we have identified four essential variables for our analyses. These variables were selected for their lower mutual correlation and considered as potentially influential variables for our study (Table 2, Fig. A1 in the Appendix).

**Table 2:** A table of raw bands and vegetation indices, considered as potentially influential for explaining bird species richness, that were used for calculating predictors in our models.

Code	Band	Wavelength (micrometers)	Rationale for including the variable
B3	Green	0.53-0.59	Play an important role in monitoring water and vegetation information.
B5	Near Infrared (NIR)	0.85-0.88	Sensitive to structural characteristics of vegetation, providing information about canopy structure and overall plant health.
**Code**	**Vegetation index**	**Formula**	
NDVI	Normalized Difference Vegetation Index	NDVI = (NIR – Red) / (NIR + Red)	Associated with the health and density of vegetation.
MNDWI	Modified Normalized Difference Water Index	(Green – SWIR) / (Green + SWIR)	Designed to enhance open water features. It also diminishes built-up area features that are often correlated with open water in other indices.

From individual bands and vegetation indices, three indicators of spectral heterogeneity were calculated from all pixels within each sampling unit (i.e., individually for each large and small square) (Fig. 4): (1) *coefficient of variation* (CV) (Gholizadeh et al., 2018, Levin et al., 2007, Mpakairi et al., 2022, Torresani et al., 2019), (2) *standard deviation* (StDev) (Warren et al., 2014), and (3) *Rao's Q index* (Rocchini et al., 2017; Torresani et al., 2019) (Table A1 in the Appendix). Rao's Q index is a recently proposed measure of spectral heterogeneity that can be applied to remote-sensed data (Rocchini et al., 2021a). It measures the distance between pixel values in spectral space, as well as their evenness. As a result, the higher diversity is related to the relative distance of spectral values and the evenness of their distribution. We used the rasterdiv R package to perform these computations (Rocchini et al., 2021b). The moving window size was set to equal the size of the squares. Unfortunately, due to the high computational cost, we were not able to compute the Raós Q index for images based on a 30 m resolution.

Additionally, we computed the median values of individual bands and vegetation indices for each square to compare them with the spectral heterogeneity metrics and to incorporate the information about landscape composition into our models. Based on these steps, we obtained 8 predictors for each of the metrics ((2 bands + 2 vegetation indices) * 2 months) for further analysis (see Fig. 4, Table A1). Their correlation structure as well as correlation with the response variable are shown in Figs. A7-9 in the Appendix.

### 2.5 Landscape types

To evaluate the effectiveness of predicting bird species richness with unclassified remote sensed data across the entire area of the Czech Republic with various types of landscape, we decided to incorporate information about the landscape type in our models. The characterization of the landscape type was based on the predominant Corine land cover class (Table 1) observed within each square. This approach was inspired by the findings by Perrone et al. (2023) or Schmidtlein et Fassnacht (2017), who concluded that SVH does not apply uniformly across different landscapes. The individual square classification into a particular landscape type was treated as a categorical variable in subsequent analyses with unclassified remote-sensed data. Because urban areas are known to play a significant role in influencing species distributions, and since there was only a single large square in which this landcover type was predominant, we made the decision to categorize an area as 'urban' even when it served as the second predominant land cover type. This choice was made to ensure that valuable information regarding urban areas is not lost in our models.

### 2.6 Statistical analysis

All statistical analyses were performed using R version 4.2.1 (R Core Team, 2021). We fitted generalized linear models (GLMs) for both large and small squares. GLMs are appropriate for count data such as species richness (studied here) and can be fitted with the appropriate error distribution and link function (e.g., Poisson or negative binomial) (Lopatin et al., 2016). We fitted GLMs using several predictor sets, log link function, and assuming a Poisson data distribution. After fitting the models, we checked for overdispersion. While for the large squares, the models were not overdispersed (the mean ± SD dispersion parameter was 1.06 ± 0.08), the models on the small squares did suffer from the overdispersion (the mean ± SD dispersion parameter was 3.71 ± 0.11). Therefore, we used the negative-binomial models for the small squares.

**Figure 5:**
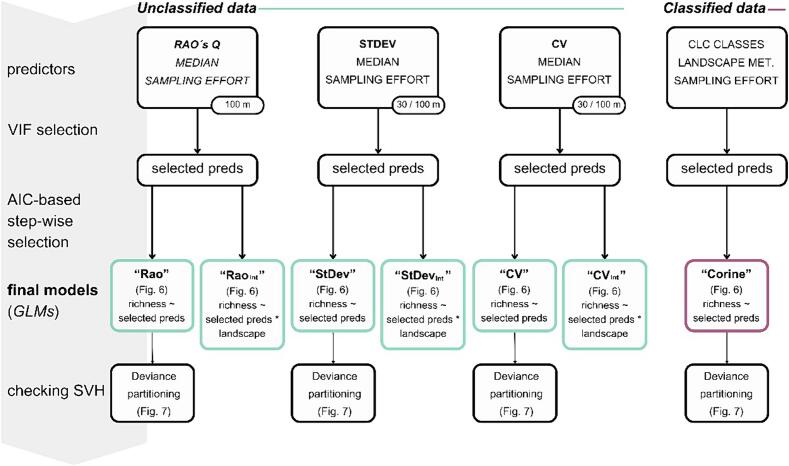
Workflow of our statistical analyses. Three predictor groups were formulated for unclassified data based on spectral heterogeneity metrics and one group for Corine data. All procedures were done for both large and small squares. We fitted 2 models within each group of predictors: a foundational model and a model incorporating landscape type in interactions. This methodology was replicated for predictor groups CV and StDev, extending the analyses to include predictors derived from raster data at a 30 m resolution.

Initially, we fitted 4 foundational models. In the first model, we tested the performance of *classified data*, i.e., Corine classes and landscape metrics. Subsequently, we generated three models based on *unclassified data* by combining median values of individual bands and vegetation indices and one of the spectral heterogeneity metrics. In addition, we included sampling effort as a covariate in all four models. To mitigate multicollinearity, we computed the variance inflation factor (VIF) for each group of predictors that entered these models. We utilized the vifcor() function (Naimi, 2017) to exclude highly correlated variables (correlation threshold set at 0.7) through a stepwise procedure. To eliminate non-significant predictors, backward stepwise selection (the Stats package (R Core Team, 2021)) was applied to all 4 models (Fig. 5). The procedure was based on the Akaike information criterion (AIC; Akaike, 1974).

A similar procedure was used to investigate the relationship between spectral heterogeneity and bird diversity across various landscape types. In the 3 full “spectral heterogeneity” models, we let all variables interact with the landscape type. The subsequent steps followed the same approach, employing stepwise selection.

### 2.7 Deviance partitioning

In order to assess the importance of individual groups of predictors, as opposed to the importance of individual predictors, we conducted deviance partitioning (Carrete et al., 2007, Peres-Neto et al., 2006). Specifically, for models testing unclassified data, we partitioned residual deviance (of species richness) from a null model (GLM, see above) with no predictors to (1) a fraction explained by the “spectral heterogeneity” predictors, (2) fraction explained by the median predictors as well as (3) by the sampling effort, (4) their overlap, and (5) a fraction explained by their independent effect. For models testing classified data, we followed the same procedure for (1) Corine class data, (2) landscape metrics, (3) sampling effort, (4) their overlap, and (5) a fraction explained by their independent effect. This method attempts to partition or resolve the explanatory power of different explanatory matrices in relation to the same response matrix (Borcard et al., 1992) and is especially useful when there is some collinearity in the predictors, or when complex hypotheses need to be tested that involve whole sets of predictors.

Our objective was also to partition explanatory power between classified and unclassified predictors. We constructed an additional model combining classified and unclassified predictors from the best-performing models (Corine and StDev models in 100 m res.) . It is important to note that in this model, we didńt focus on explaining the variability in species richness, nor did we address issues related to data collinearity. Rather, the primary aim was to construct a model that effectively illustrates the partitioning dynamics between these distinct categories of predictors.

### 2.8 Spatial autocorrelation

To address the potential presence of spatial autocorrelation effects in our study, we utilized Moran's correlogram to measure autocorrelation in species richness. We examined data from both small and large squares and observed that autocorrelation was minimal in small squares (max. Moran's I: 0.07), while a weak autocorrelation was observed in the large squares (max. Moran's I: 0.20). To address this, we conducted an analysis of residuals from our models, which further supported the absence of substantial autocorrelation effects in our research outcomes (max. Moran's I: 0.06 for small squares and max. Moran's I: 0.09 for large squares) (Figs. A2 and A3 in the Appendix).

## 3 Results

### 3.1 Scale

**Figure 6:**
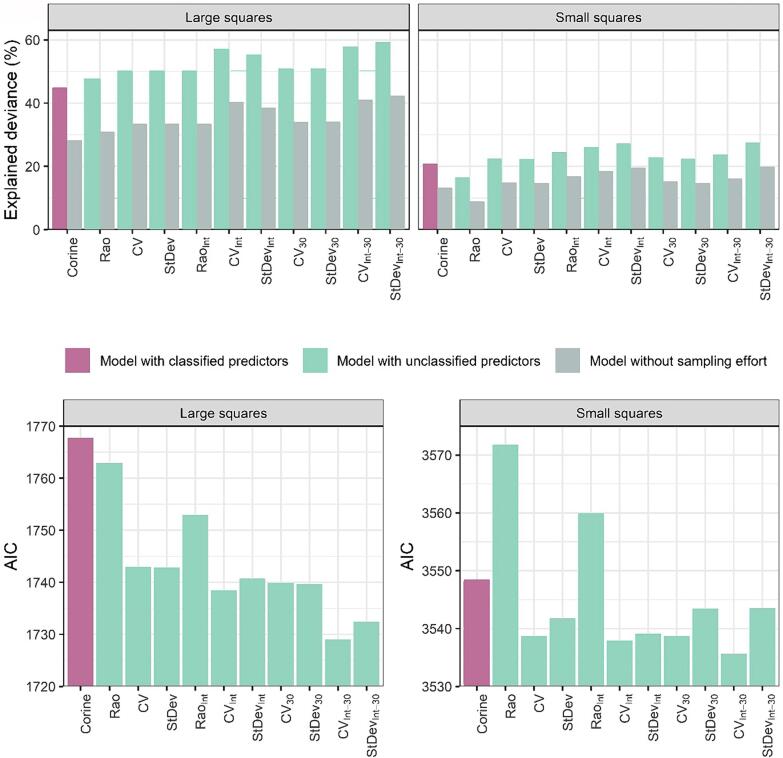
Explained deviance (upper panels) and AIC values (lower panels) for the final GLMs fitted on data from large and small squares. Apart from the overall explained deviance values (pink and green columns in the upper panels), the explained deviance reduced by the part explained solely by sampling effort is shown (grey columns in the upper panels).

We considered scale in two ways, namely *1) the original image resolution (pixel size)* and *2) grain (sampling unit)*. In terms of AIC, the models based on 30 m resolution slightly outperformed the ones based on 100 m resolution in case of large squares. The reduction of AIC was about 3 and 9 points for models with and without interactions with landscape types, respectively (Fig. 6). For small squares, the AIC values were more stable, not favoring any of the two resolutions. The average variability in bird species richness explained by all models was similar when comparing a 100 m to a 30 m resolution (Fig. 6). In models without interactions with landscape type, the average explained deviance for large squares was 48% and 51% for models based on a 100 m and 30 m resolution, respectively. For small squares, these values were 21% and 23% for a 100 m and 30 m resolution, respectively. In models with interactions, the average explained deviances for large squares were 54% and 58% for the 100 m and 30 m resolutions, respectively while for small squares, it was 26% for both resolutions.

The differences between the results from the two grains were more pronounced. The overall average explained deviance was 52% (SD: 4.5) for large squares and 23% (SD: 3.1) for small squares, respectively. Due to the significantly weaker results for small squares and little differences between the models in terms of the original raster resolution, subsequent results from our study (emphasizing the predictors effects, deviance partitioning etc.) will be mostly presented for models based on large squares at a 100 m resolution raster. The coefficients of all the models are summarized in the Tables A2-10 in the Appendix. The explained deviance and AIC values are shown in Fig. 6. The observed vs predicted plots are in the Figs. A4 and A5 in the Appendix.

### 3.2 Models using classified predictors

**Figure 7:**
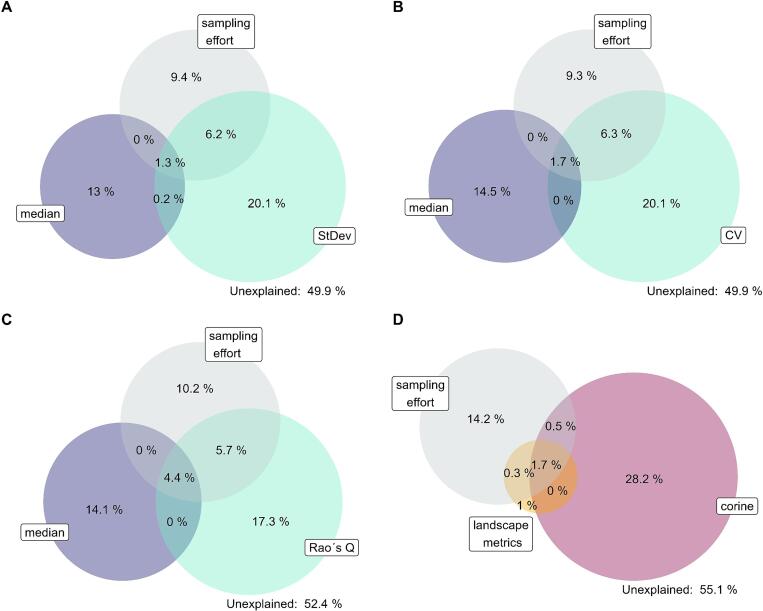
Deviance partitioning for models based on unclassified data in large squares at a 100 m resolution (A - C) and on classified data in large squares (D). In the models based on unclassified data, the spectral heterogeneity was measured by Rao’s Q (A), coefficient of variation (B), and standard deviation (C). Each color represents one group of predictors used in the model. The overlap of the bubbles indicates shared variability explained by a given combination of predictors. The remaining unexplained variability is also specified.

Models fitted with Corine predictors captured 45% of the bird species richness variability in large squares and 21% in small squares. Despite the substantial explained variability in large squares, the AIC was high compared to other models (Fig. 6). After the exclusion of the part explained by sampling effort only, the explained variability decreased to 28% and 13 % for large and small squares, respectively (the grey columns in Fig. 6). Furthermore, the model with unclassified predictors also showed a notable bias, overestimating the species richness in most of the squares (Figs. A4 and A5 in the Appendix). There is a small overlap among variability explained by all groups of predictors (Fig. 7).

In the models based on Corine predictors on large squares, the largest effect on species richness was exhibited by the extent of urban areas (-47 species over the predictor range), sampling effort (+44 species over the predictor range) and the area of water (+35 species over the predictor range) (Table A2, Fig. A9 in the Appendix). The effects of other predictors were considerably weaker (Fig. A9).

### 3.3 Models using unclassified predictors

Models with unclassified predictors were able to explain up to 51% of the variability in bird species richness in large squares, and up to 23% in small squares (Fig. 6), a part of which, however, could be explained by the sampling effort. After deduction of the variability explained solely by the sampling effort, we obtained the net variability explained by unclassified predictors, which was 33 % and 15 % for large and small squares, respectively. The coefficient of variation (CV) and standard deviation (StDev) yielded equivalent results, while Rao’s Q index exhibited a slightly inferior performance in terms of explained deviance and markedly higher AIC value (Fig. 6). For both large and small squares, spectral heterogeneity metrics were the most powerful predictors. The overlap between spectral heterogeneity and median predictors is consistently small in all models. Besides, there is no overlap between median values and sampling effort. Additionally, some of the variability explained by spectral heterogeneity is shared with the variability explained by sampling effort (Fig. 7).

All three models for large squares that were based on unclassified predictors yielded almost the same significant predictors; these similarities were especially obvious between the models with CV and StDev heteronegeity metrics (Tabs. A3, A5 in the Appendix). From the heterogeneity predictors, the April MNDWI was the most important predictor, showing an increase of 45 species over its range for the model with StDev (Fig. A10 in the Appendix). The median of the green band reflectance exhibited an even stronger effect (+52 species), with sampling effort also showing a strong effect (+33 sp.; see Fig. A10 for all effects).

### 3.4 Models using unclassified predictors interacting with landscape types

When including the information about the environment (landscape type), our models showed a reduction in AIC by up to 10 points, and they explained an even greater portion of variability – up to 57% in large squares and up to 27% in small squares, respectively (up to 40% and 20% after the exclusion of sampling efforts) (Fig. 6).

The selection of significant predictors not interacting with landscape types as well as their effects were almost identical as for the models without interactions (compare Tables A3, A5 with the Tables A4, A6, and Fig. A10 with Fig. A11 in the Appendix). For the models based on the StDev heterogeneity metric, the heterogeneity of the April NIR band represented the only significant interaction with landscape types. The effect of this interaction was positive in the areas dominated by agriculture or coniferous forests, while it was negative in the leaved-and-mixed forests and urban areas (Fig. A11).

### 3.5 Comparison of classified vs unclassified predictors

**Figure 8:**
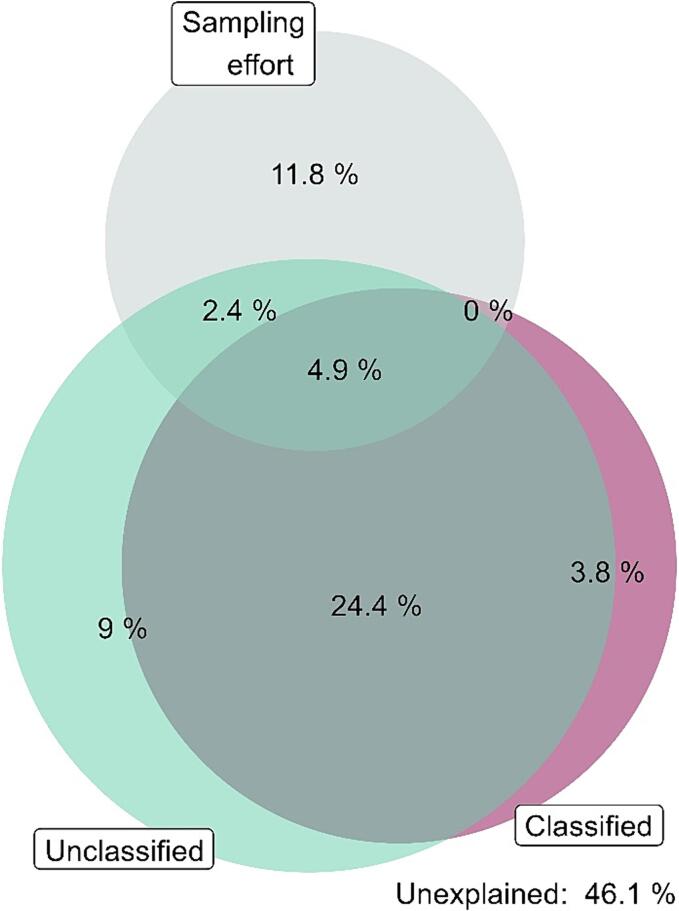
Deviance partitioning for the model combining unclassified data, classified data, and sampling effort as predictors. The unclassified predictors are those from the best performing model with unclassified data, i.e. the model for large squares based on 100 m resolution rasters using standard deviation as a metric of spectral heterogenenity. The Corine predictors are based on the model with Corine predictors for large squares. Each color represents one group of predictors used in the model. The overlap of the bubbles indicates the shared variability explained by a given combination of predictors. The remaining unexplained variability is also specified.

The model combining predictors based on classified and unclassified remote-sensed data (StDev and median values) explained 54% of the variability in species richness; nevertheless, a large fraction of explained variability cannot be attributed to any individual group of predictors (Fig. 8). The spectral heterogeneity predictors explained the largest portion of variability; still, although the results are better than those yielded by models based on Corine predictors the performance of the latter was slightly inferior.

**Figure 9:**
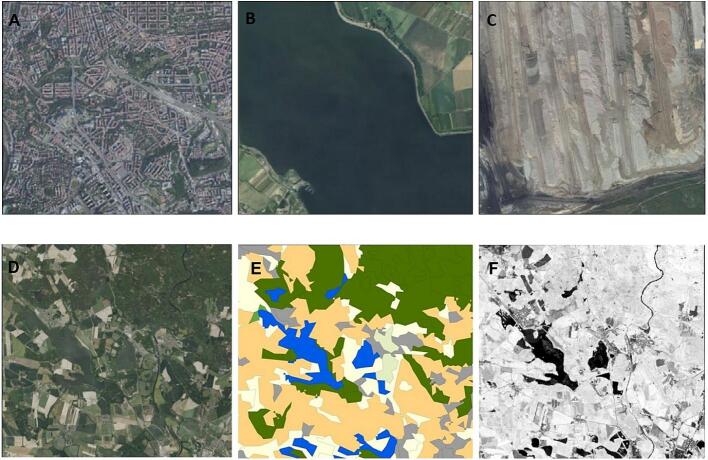
Examples of small squares filled with mostly one type of environment, but with high spectral heterogeneity (A: city center, B: water body, C: surface mine); Another example: large squares with high spectral variability and also high species richness, D: orthophoto map, E: Corine land cover classes, F: May NDVI.

## 4 Discussion

In our study, we have demonstrated that models based on unclassified remote-sensed data, particularly metrics representing spectral heterogeneity, can explain the variability in bird species richness better compared to models and metrics based on classified land cover data. As classified remote-sensed data are only available from certain regions, the possibility of using unclassified data that are more widely available is good news for researchers focusing on such areas. Nevertheless, the model combining all types of data showed that the shared explained variability was substantial, indicating that both types of data contain similar information about the environment.

Putting aside the question of scale/spatial extent, our findings can be compared with those published by Duro et al. (2014) or Ribeiro et al. (2019), who found that predictors derived from continuous information (NDVI) were consistently better predictors of species diversity than predictors derived from discrete classifications. In contrast, Culbert et al. (2012) found that bird species diversity in Midwestern USA was slightly better explained by landscape composition metrics based on land cover classification than by predictors based on continuous spectral information (image texture); however, they also state that the satellite images used in this study were not acquired at the same phenological stage, which may have likely introduced extraneous variability into texture measurements. The amount of variability in species richness explained by our models is comparable to other similar studies based on Landsat imagery (Culbert et al., 2012, Duro et al., 2014, Hunt et al., 2022, Levin et al., 2007, Rocchini, 2007, Wood et al., 2013).

We had expected that effects of environmental predictors would be influenced by the type of landscape in which the analysis was done. Many studies were conducted over small geographical extents or typically focused on a single habitat type (Bino et al., 2008, Duro et al., 2014, Goetz et al., 2010, St-Louis et al., 2014, Wood et al., 2013). Compared to these, we included a variable that characterizes various landscape types, and let it statistically interact with other predictors in the models. Surely enough, including the interaction explained more variation in our models and led to a reduction in AIC. This is consistent with Perrone et al. (2023) or Schmidtlein et Fassnacht (2017) who concluded that SVH does not hold uniformly across different landscape types. Ludwig et al. (2024) also emphasized that the applicability of SVH is not universal; it varies not only across ecosystems but also across seasons and sensors.

We showed that spectral heterogeneity can explain a significant portion of the variability in bird species richness; this link also depended on specific metrics characterizing spectral heterogeneity. We compared three metrics of spectral heterogeneity: the coefficient of variation (CV), standard deviation (StDev), and Rao's Q index. The CV and StDev metrics were almost identical, both in terms of model fit and predictor effects. A single predictor – the heterogeneity of April MNDWI – played a major role in explaining the species richness in these models. Models using Rao's Q predictors differed in terms of predictor selection and also exhibited weaker performance. The long computation time and the inability to calculate it at a 30-meter resolution were additional limitations of Rao's Q. Based on other studies (Bino et al., 2008, Sheeren et al., 2014, St-Louis et al., 2014), we expected that higher species richness would be associated with sites showing higher spectral heterogeneity; however, the effect of spectral heterogeneity was sometimes negative, especially in interactions with certain landscape types. We, therefore, suggest that the direction of correlation (positive or negative) between species richness and spectral heterogeneity is likely to be influenced by the type of landscape.

Besides the indices of spectral heterogeneity and type of landscape, scale is another important factor (Moudrý et al., 2023a). In our study, we considered two types of scale: we compared (a) predictors derived from images with 30 m and 100 m resolutions (pixel sizes), as well as (b) two different sizes of mapping units (grain). Rocchini (2007) claimed that a decrease in resolution (pixel size) would decrease the predictive ability of spectral heterogeneity because of more mixed reflectance within a pixel. However, we observed almost no improvement in models based on images with 30 m resolution. According to our results, the size of the mapping unit (large and small squares, in our case) plays a more important role in explaining species diversity than the original resolution of the imagery (see Fig. 6).

Based on other studies (Betts et al., 2007, Culbert et al., 2012, St-Louis et al., 2014), we expected models using unclassified data for small squares to perform better than those for the large squares. Surprisingly, this was not the case, and the amount of explained variability in species richness was lower in the small squares than in the large ones. This observed poor relationship between species richness and spectral heterogeneity in small squares may be explained by the fact that small squares are more prone to containing only a single landscape type (Fig. 9) such as surface mines, city centers, and mountains. These have high spectral heterogeneity but low bird diversity, and, therefore, SVH fails in squares dominated by these landscape types. In contrast, large squares contain more landscape types, including species-rich types such as forests, and, thus, the spectral heterogeneity within these squares is more biologically relevant. An additional explanation is ecological: the finer the resolution, the higher is the chance of stochastic and neutral processes driving community assembly and diversity (Leibold and Chase, 2017) to compromise the SVH validity. The demographic stochasticity (Lande et al. 2010), which emerges from random variation in fecundity and mortality at an individual level at local scales and which can lead to local extinctions, can be used as an example. Stochastic source-sink metapopulation dynamics (Hanski 2001) can be used as another example – it is detectable as a fluctuating occupancy at fine grains, but as stable occupancy at coarse grains. Both these examples can lead to the weakening of the deterministic species-environment correlations towards local grains. Inversely, the coarser the resolution, the stronger the effect of environmental deterministic processes on diversity, which is a pattern with strong empirical support across other taxonomic groups and regions (Field et al., 2009).

Additionally, in Corine models, we also noted a lower performance for small squares. We hypothesize that this may be attributed to the spatial scale of Corine data, which has a minimum mapping unit of 0.25 km^2^. This coarse spatial resolution could be inadequate when applied to the scale of small squares.

We are aware that our study comes with limitations. Although we used data from systematic sampling, these data exhibit some biases typical of citizen science. Several factors have been described as strongly influencing the sampling effort, such as the presence of roads, water bodies, and urban areas (Engemann et al., 2015, Zizka et al., 2021). When comparing models for classified and unclassified data, distinct patterns emerge regarding the influence of sampling effort. In models utilizing Corine data, the shared deviance explained between Corine predictors and sampling effort is minimal. Conversely, in models with unclassified data, there is an observable shared explained deviance between spectral heterogeneity and sampling effort. This observation suggests that there is also a relationship between the sampling effort and environmental heterogeneity, suggesting preferences of people to visit more heterogeneous landscapes.

## 5 Conclusion

We have demonstrated that unclassified remote-sensed data from Landsat 8 can explain a similar or greater amount of spatial variability of bird species richness than predictors derived from classified land cover data such as Corine. We showed that spectral heterogeneity is a good predictor of bird species richness, but we do not provide ultimate support for the SVH – whether the relationship between spectral heterogeneity and species richness is positive or negative depends on the landscape type. We found that the size of the mapping unit plays a more important role in explaining species diversity than the original resolution of the imagery. The use of unclassified remote-sensed data as a straightforward and efficient predictor of bird diversity holds potential. This approach can enhance our understanding of the environmental associations with higher species diversity and inform decision-making authorities concerning biodiversity conservation.

## CRediT authorship contribution statement

**Dominika Prajzlerová:** Writing – review & editing, Writing – original draft, Methodology, Investigation, Formal analysis. **Vojtěch Barták:** Writing – review & editing, Methodology, Investigation. **Petr Keil:** Writing – review & editing, Writing – original draft, Methodology. **Vítězslav Moudrý:** Writing - Original Draft, Supervision. **Markéta Zikmundová:** Formal analysis. **Petr Balej:** Software. **François Leroy:** Writing – original draft. **Duccio Rocchini:** Writing – original draft, Supervision. **Michela Perrone:** Writing – original draft, Validation. **Marco Malavasi:** Writing – original draft. **Petra Šímová:** Writing – original draft, Supervision, Methodology, Conceptualization.

## Declaration of competing interest

The authors declare that they have no known competing financial interests or personal relationships that could have appeared to influence the work reported in this paper.

## Data Availability

The authors do not have permission to share data.
