# Supplementary material for: The relationship between remotely-sensed spectral heterogeneity and bird diversity is modulated by landscape type
Source: Int J Appl Earth Obs Geoinf. 2024 Apr;128:103763. doi: 10.1016/j.jag.2024.103763 (PMC11004726; doi:10.1016/j.jag.2024.103763)
Supplement: Supplementary data 1 [file mmc1.docx]

## Appendix

**Table A1.** List of all, unclassified and classified, predictors utilized in the analysis.

| **Unclassified predictors** |  | **Classified predictors** |  |
| --- | --- | --- | --- |
| *Median* | B3_april_median | *Area of CLC classes* | Artificial surfaces |
|  | B3_july_median |  | Coniferous forests |
|  | B5_april_median |  | Broad-leaf and mixed forests |
|  | B5_july_median |  | Open vegetation |
|  | NDVI_april_median |  | Arable land and permanent crops |
|  | NDVI_july_median |  | Pastures and heterogenous agricultural areas |
|  | MNDWI_april_median |  | Wetlands and water bodies |
|  | MNDWI_july_median | *Landscape metrics* | Patch richness |
| *Standard deviation* | B3_april_stdev |  | Number of patches |
|  | B3_july_stdev |  | Largest patch area |
|  | B5_april_stdev | *Landscape types* | Predominant area |
|  | B5_july_stdev |  |  |
|  | NDVI_april_stdev |  |  |
|  | NDVI_july_stdev |  |  |
|  | MNDWI_april_stdev |  |  |
|  | MNDWI_july_stdev |  |  |
| *Coefficient of variation* | B3_april_cv |  |  |
|  | B3_july_cv |  |  |
|  | B5_april_cv |  |  |
|  | B5_july_cv |  |  |
|  | NDVI_april_cv |  |  |
|  | NDVI_july_cv |  |  |
|  | MNDWI_april_cv |  |  |
|  | MNDWI_july_cv |  |  |
| *Rao´s Q index* | B3_april_rao |  |  |
|  | B3_july_rao |  |  |
|  | B5_april_rao |  |  |
|  | B5_july_rao |  |  |
|  | NDVI_april_rao |  |  |
|  | NDVI_july_rao |  |  |
|  | MNDWI_april_rao |  |  |
|  | MNDWI_july_rao |  |  |

**Table A2**. Metrics for spectral heterogeneity, their explanations, and the research studies in which they have been employed.

| **Heterogeneity metric** | **Description** | **Research studies** | **Computational requirements** | |
| --- | --- | --- | --- | --- |
| *Standard deviation* | quantifies the degree of variation or spread in spectral values within a dataset | Hall et al. (2010)  Palmer et al. (2002)  Perrone et al. (2023) | | simple |
| *Coefficient of variation* | indicates the relative amount of variation in the dataset compared to its mean | Levin et al. (2007)  Mpakairi et al. (2022)  Oindo et Skidmore (2002) | | simple |
| *Rao´s Q index* | assesses heterogeneity in a more complex manner by considering the pairwise spectral dissimilarities among pixels or sample points within a dataset | Michele et al. (2018)  Torresani et al. (2019)  Rocchini et al. (2018) | | intensive |


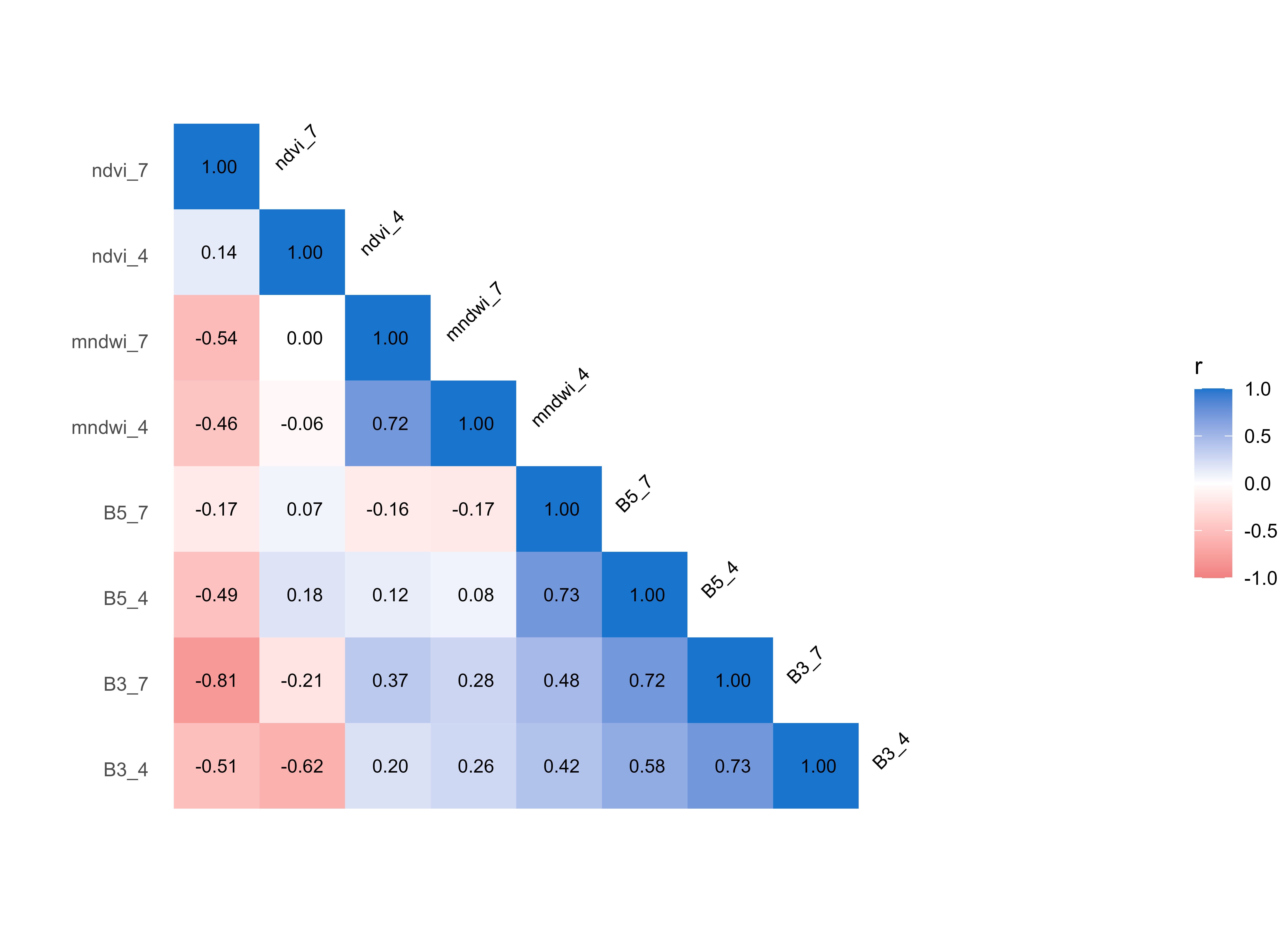


**Figure A1.** Correlation matrix between rasters derived for raw bands and vegetation indices for April (4) and July (7).


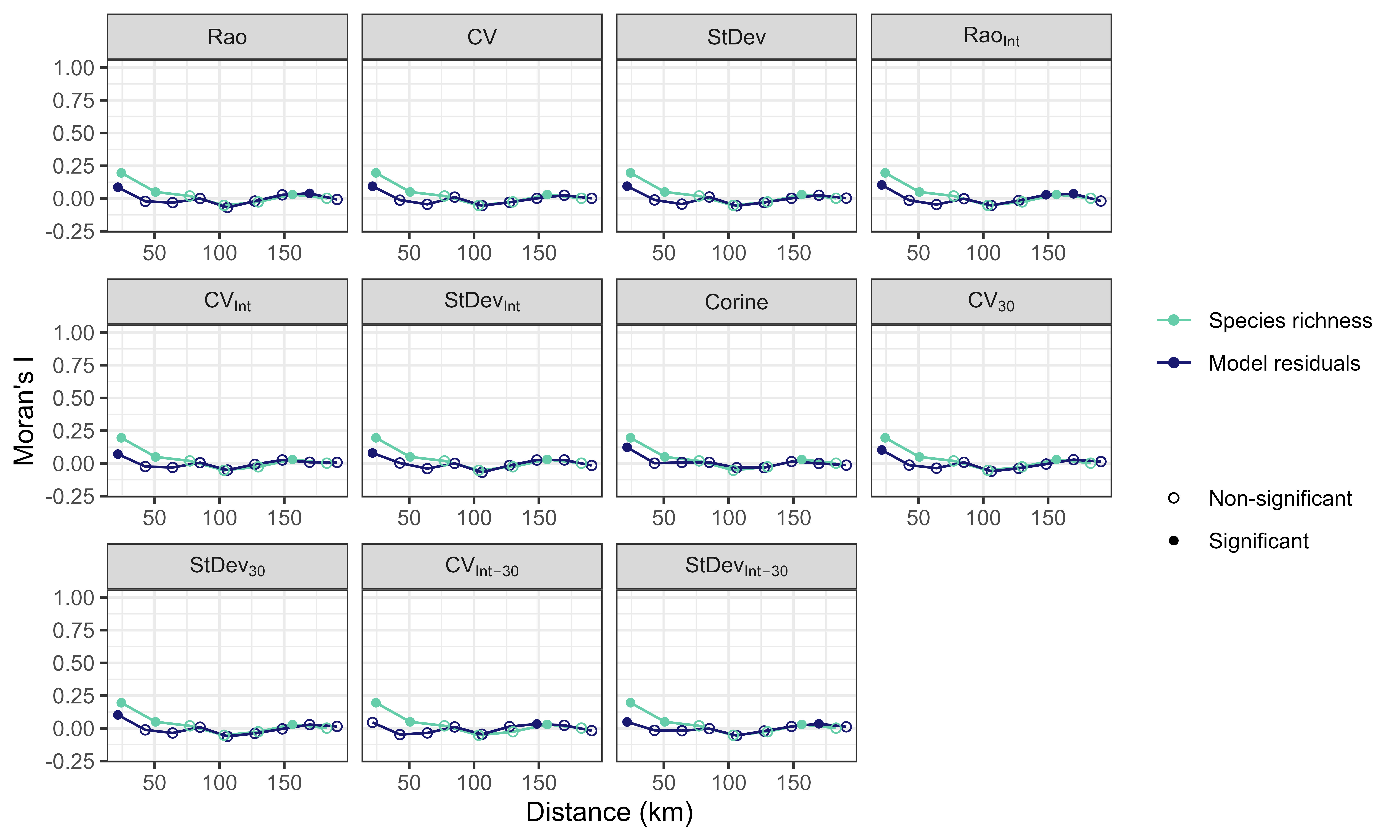


**Figure A2**. Large squares: the correlograms illustrate spatial autocorrelation patterns. The light blue line represents the autocorrelation for species richness, while the dark blue line represents the autocorrelation for model residuals in each model.


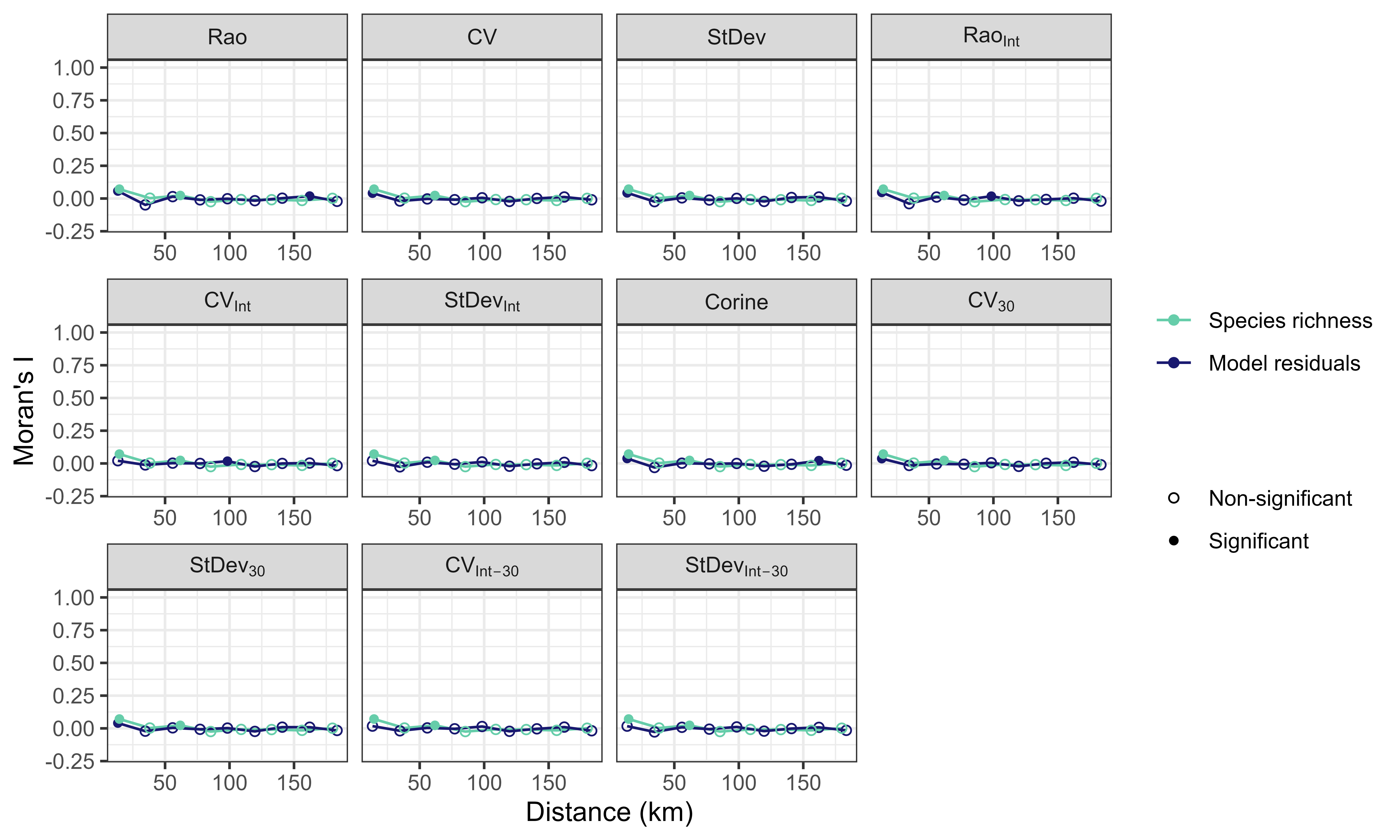


**Figure A3**. Small squares: the correlograms illustrate spatial autocorrelation patterns. The light blue line represents the autocorrelation for species richness, while the dark blue line represents the autocorrelation for model residuals in each model.


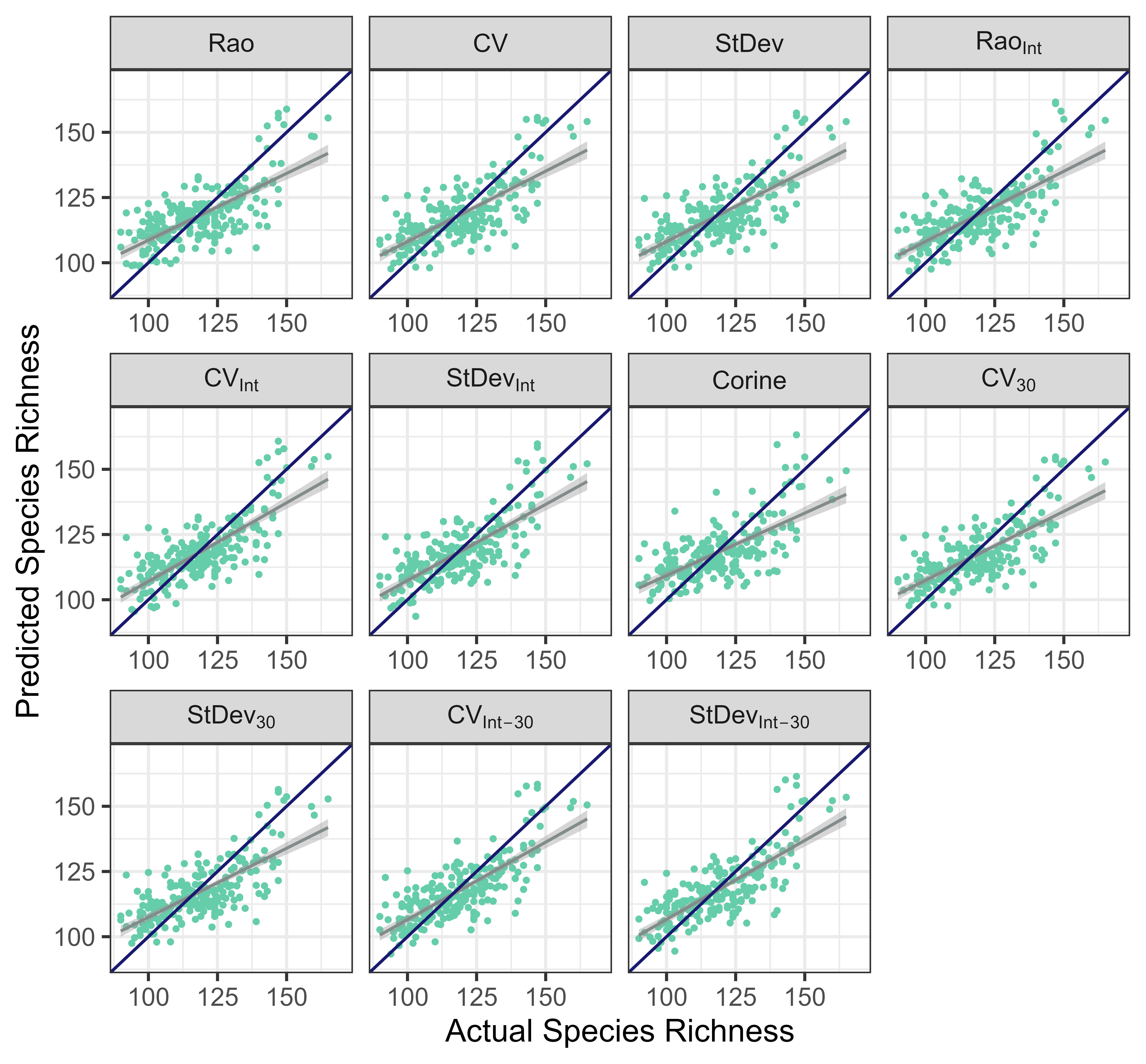


**Figure A4.** Large squares: predicted vs. actual species richness. The blue line depicts the first quadrant axis, the gray line represents the linear trend, along which the 95% confidence bands are shown.


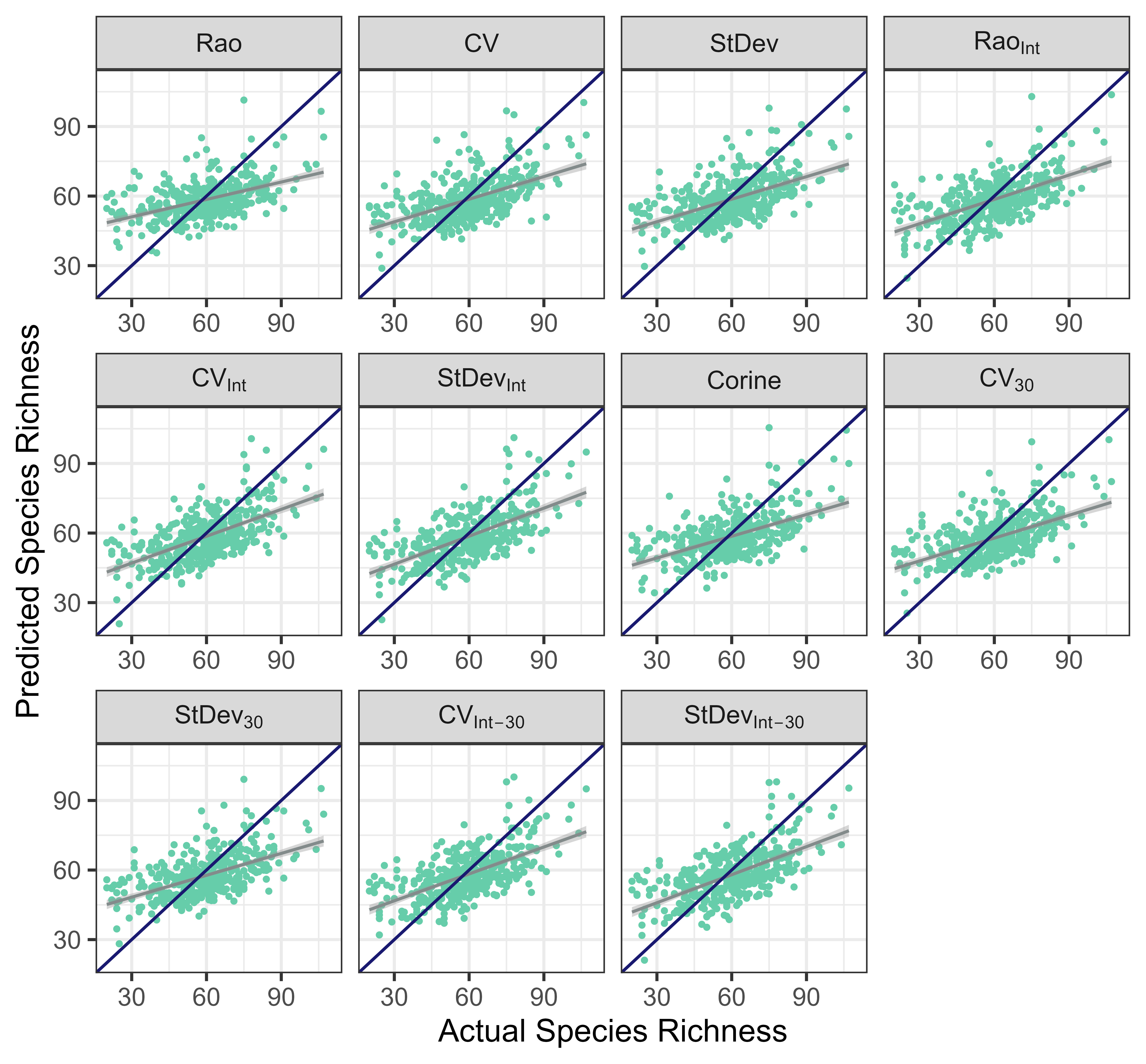


**Figure A5.** Small squares: predicted vs. actual species richness. The blue line depicts the first quadrant axis, the gray line represents the linear trend, along which the 95% confidence bands are shown.


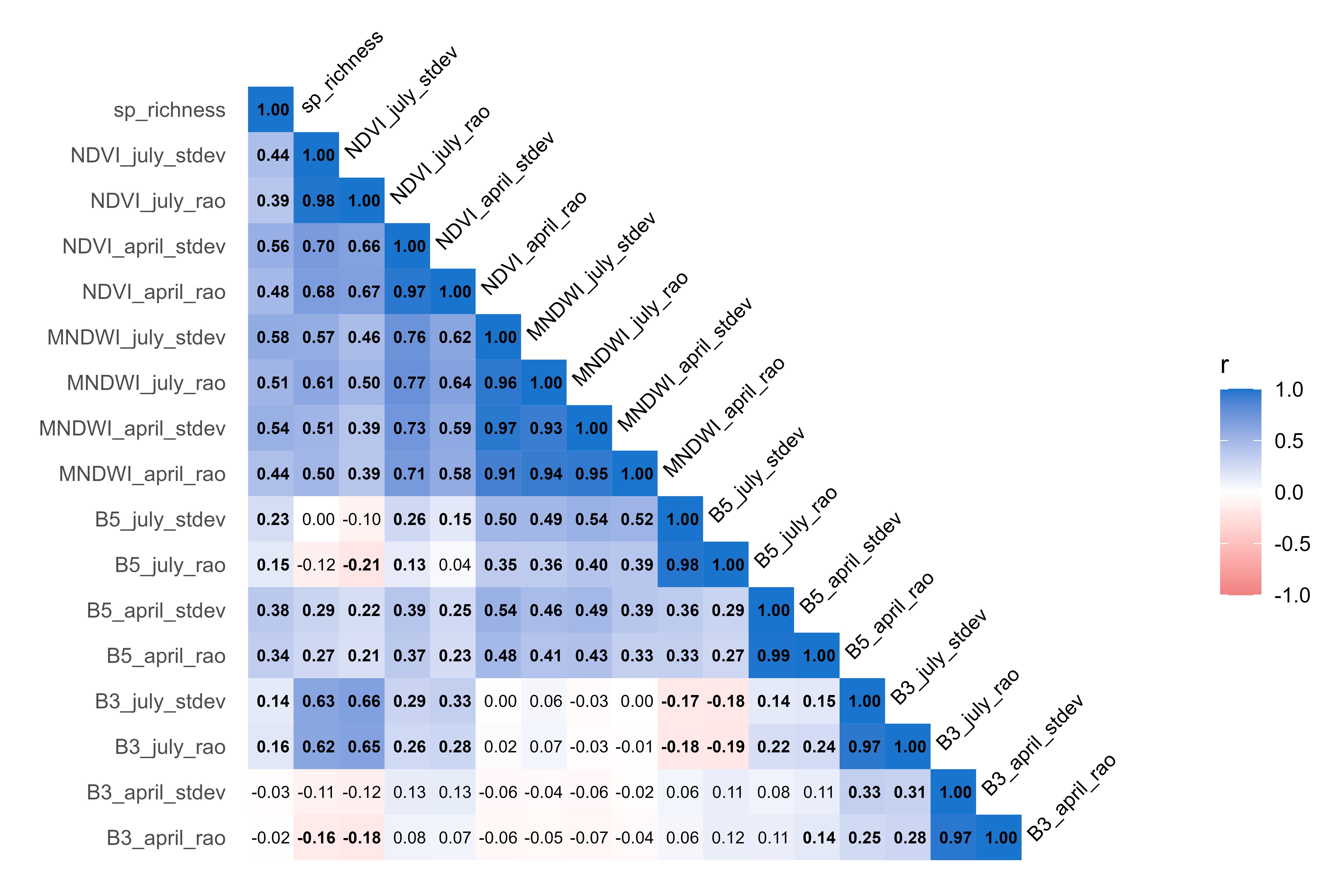


**Figure A6.** Correlation matrix between Rao´s Q and StDev predictors and species richness for large squares. The significant values are presented in bold.


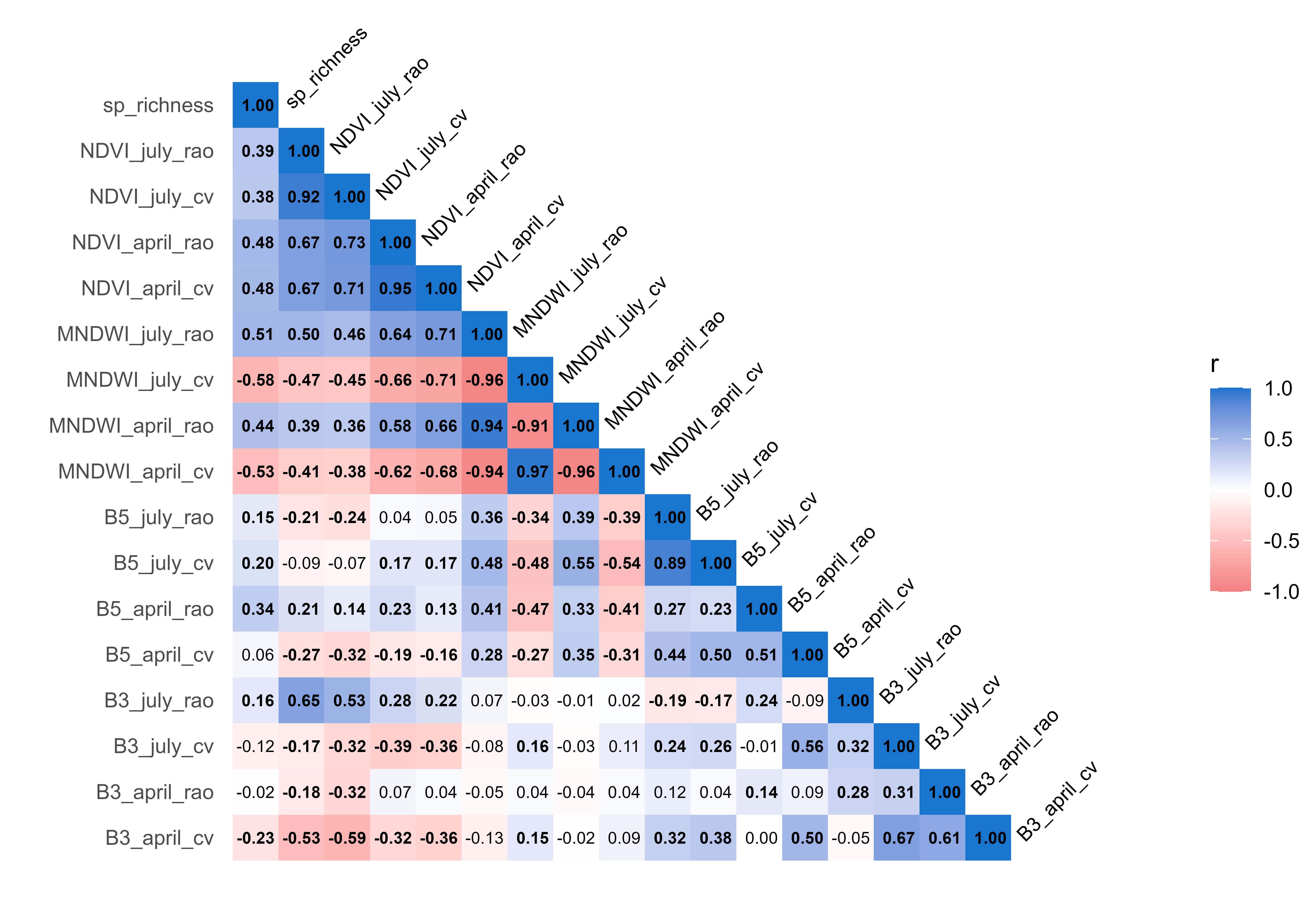


**Figure A7.** Correlation matrix between Rao´s Q and CV predictors and species richness for large squares. The significant values are presented in bold.


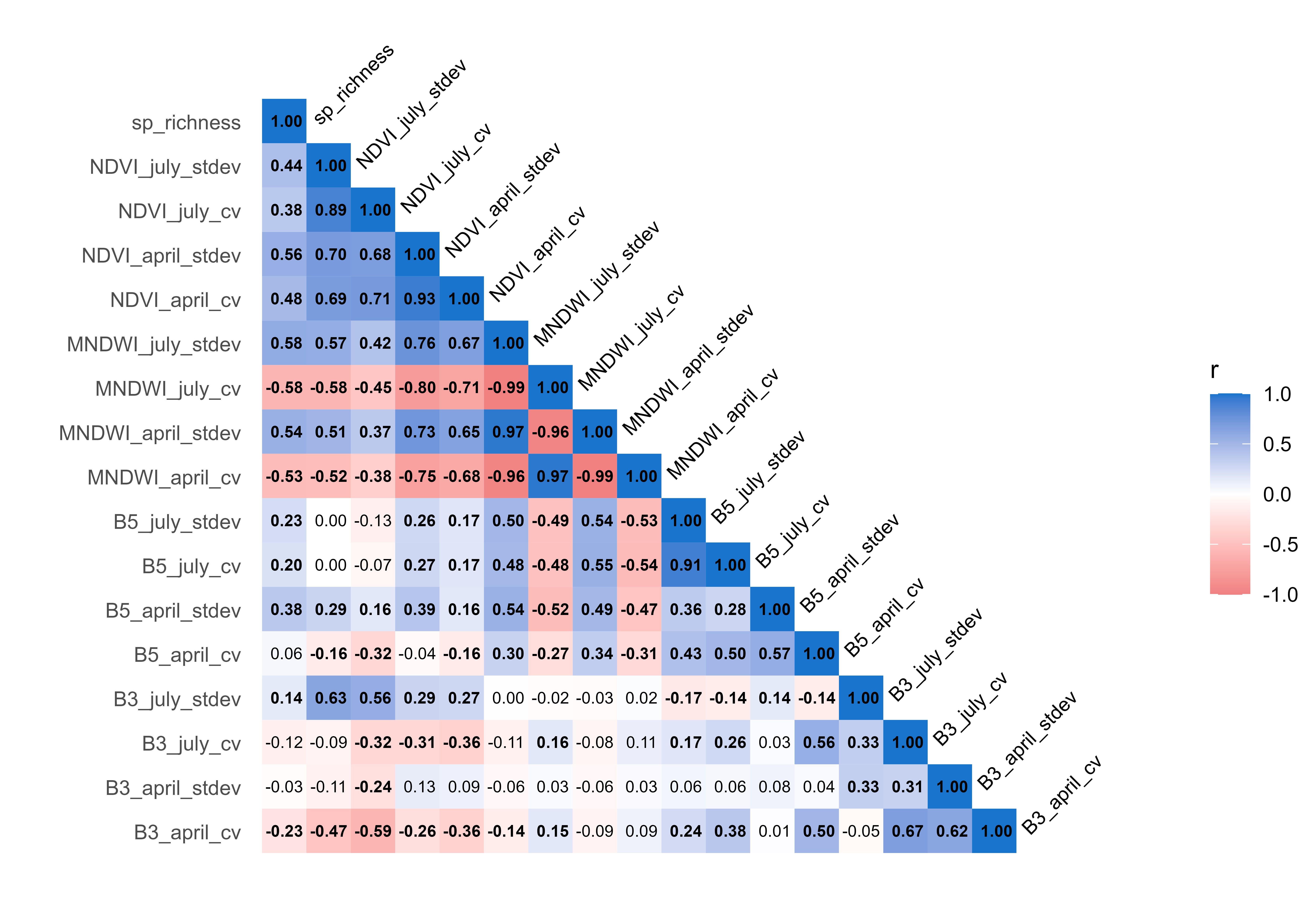


**Figure A8.** Correlation matrix between CV and StDev predictors and species richness for large squares. The significant values are presented in bold.

**Table A3.** Coefficients, their standard errors (in the parentheses), and significance (asterisks) of individual model terms of the final GLMs using classified Corine predictors. Significant values are bold, with the significance level coded as follows: < 0.001 ‘***’, < 0.01 ‘**’, < 0.05 ‘*’.

| **Classified predictors** | | |
| --- | --- | --- |
| **Model term** | **Model on large squares** | **Model on small squares** |
| Dev. explained, AIC | 45%, 1767.7 | 21%, 3548.5 |
| Intercept | **4.6826 (0.03928)***** | **3.7615 (0.04712)***** |
| *Area of individual Corine classes* | | |
| Coniferous | **-0.0014 (0.00036)***** | - |
| Leaves and mixed | **-0.0023 (0.00043)***** | **0.0174 (0.00548)**** |
| Urban | **-0.0044 (0.00072)***** | **-0.0393 (0.00551)***** |
| Water | **0.015 (0.00224)***** | **0.1322 (0.018)***** |
| Agro | - | **0.0195 (0.00483)***** |
| Natural agro | - | **0.0568 (0.00774)***** |
| Open vegetation | - | **0.0423 (0.00967)***** |
| *Landscape metrics* | | |
| Patch richness | **0.0072 (0.00331)*** | **0.0293 (0.00524)***** |
| Number of patches | - | **-0.0065 (0.0016)***** |
| Largest patch area | - | **-0.0195 (0.00582)***** |
| *Sampling effort* |  |  |
| Number of visits | **0.0005 (0.00006)***** | **0.0069 (0.00042)***** |

**Table A4.** Coefficients, their standard errors (in the parentheses), and significance (asterisks) of individual model terms of the final GLMs using unclassified Landsat 8 predictors, computed from 100 m resolution rasters on large squares. Significant values are bold, with the significance level coded as follows: < 0.001 ‘***’, < 0.01 ‘**’, < 0.05 ‘*’.

| **Unclassified predictors, large squares, 100 m resolution** | | | |
| --- | --- | --- | --- |
| **Model term** | **Model with Rao** | **Model with CV** | **Model with StDev** |
| Dev. explained, AIC | 48%, 1762.9 | 50%, 1742.0 | 50%, 1742.8 |
| Intercept | **2.64 (0.243)***** | **2.99 (0.221)***** | **3.13 (0.219)***** |
| *Spectral heterogeneity* | | | |
| Green_July_ | -4.19 (2.840) | - | - |
| NDVI_April_ | **0.87 (0.392)*** | - | - |
| NDVI_July_ | 0.56 (0.322) | - | - |
| MNDWI_April_ | **0.88 (0.340)**** | **-0.54 (0.054)***** | **1.13 (0.113)***** |
| *Medians* | | | |
| Green_April_ | **8.88 (1.576)***** | **8.48 (1.134)***** | **8.36 (1.135)***** |
| NIR_July_ | **-1.22 (0.441)**** | **-0.94 (0.362)**** | **-0.94 (0.362)**** |
| NDVI_April_ | **0.86 (0.167)***** | **0.71 (0.157)***** | **0.70 (0.157)***** |
| NDVI_July_ | **0.29 (0.125)*** | - | - |
| MNDWI_April_ | **-1.89 (0.318)***** | **-1.89 (0.310)***** | **-1.63 (0.310)***** |
| *Sampling effort* | | | |
| Number of visits | **0.0004 (0.00005)***** | **0.0004 (0.00005)***** | **0.0004 (0.00005)***** |

**Table A5.** Coefficients, their standard errors (in the parentheses), and significance (asterisks) of individual model terms of the final GLMs including interactions with landscape types. Predictors computed using unclassified Landsat 8 predictors, from 100 m resolution rasters, and on large squares. Significant values are bold, with the significance level coded as follows: < 0.001 ‘***’, < 0.01 ‘**’, < 0.05 ‘*’.

| **Unclassified predictors, large squares, 100 m resolution, interactions with landscape** | | | |
| --- | --- | --- | --- |
| **Model term** | **Model with Rao** | **Model with CV** | **Model with StDev** |
| Dev. explained, AIC | 50%, 1753.0 | 57%, 1738.4 | 55%, 1740.7 |
| Intercept | **2.97 (0.260)***** | **3.17 (0.299)***** | **3.50 (0.267)***** |
| *Spectral heterogeneity* | | | |
| Green_April_ | -2.21 (1.508) | - | - |
| Green_July_ | - | -0.04 (0.167) | - |
| NIR_April_ | - | - | -1.85 (1.496) |
| NIR_July_ | - | - | 0.88 (0.934) |
| NDVI_April_ | **1.06 (0.377)**** | - | - |
| NDVI_July_ | - | 0.14 (0.134) | - |
| MNDWI_April_ | **1.01 (0.289)***** | **-0.47 (0.062)***** | **1.08 (0.193)***** |
| *Medians* | | | |
| Green_April_ | **8.98 (1.576)***** | **9.19 (1.71)***** | **7.27 (1.537)***** |
| NIR_July_ | **-0.84 (0.412)*** | **-0.94 (0.45)*** | **-0.93 (0.407)*** |
| NDVI_April_ | **0.72 (0.179)***** | **0.49 (0.217)*** | **0.54 (0.231)*** |
| MNDWI_April_ | **-1.55 (0.345)***** | **-1.67 (0.334)***** | **-1.38 (0.326)***** |
| *Landscape types* | | | |
| LT-Coniferous | **0.06 (0.023)*** | **-0.74 (0.368)*** | -0.12 (0.173) |
| LT-Leaves | 0.03 (0.037) | 1.10 (0.58) | -0.19 (0.382) |
| LT-Urban | **-0.07 (0.021)***** | -0.06 (0.265) | -0.30 (0.182) |
| *Interactions of spectral heterogeneity with landscape types* | | | |
| Green_July_: LT-Coniferous | - | 0.56 (0.312) | - |
| Green_July_: LT-Leaves | - | 0.40 (0.537) | - |
| Green_July_: LT-Urban | - | -0.48 (0.338) | - |
| NIR_April_: LT-Coniferous | - | - | **5.02 (1.906)**** |
| NIR_April_: LT-Leaves | - | - | -2.14 (3.572) |
| NIR_April_: LT-Urban | - | - | 5.02 (2.594) |
| NIR_July_: LT-Coniferous | - | - | -3.56 (1.884) |
| NIR_July_: LT-Leaves | - | - | 4.27 (3.670) |
| NIR_July_: LT-Urban | - | - | -1.86 (1.593) |
| NDVI_July_: LT-Coniferous | - | 0.32 (0.350) | - |
| NDVI_July_: LT-Leaves | - | -1.41 (0.837) | - |
| NDVI_July_: LT-Urban | - | -0.47 (0.307) | - |
| *Interactions of medians with landscape types* | | | |
| NDVI_April_: LT-Coniferous | - | 0.78 (0.566) | - |
| NDVI_April_: LT-Leaves | - | **-1.69 (0.848)*** | - |
| NDVI_April_: LT-Urban | - | 0.51 (0.402) | - |
| *Sampling effort* | | | |
| Number of visits | **0.0004 (0.00005)***** | **0.0004 (0.00006)***** | **0.0004 (0.00006)***** |

**Table A6.** Coefficients, their standard errors (in the parentheses), and significance (asterisks) of individual model terms of the final GLMs using unclassified Landsat 8 predictors, computed from 30 m resolution rasters on large squares. Significant values are bold, with the significance level coded as follows: < 0.001 ‘***’, < 0.01 ‘**’, < 0.05 ‘*’.

| **Unclassified predictors, large squares, 30 m resolution** | | |
| --- | --- | --- |
| **Model term** | **Model with CV** | **Model with StDev** |
| Dev. explained, AIC | 51%, 1739.9 | 51%, 1739.6 |
| Intercept | **3.02 (0.220)***** | **3.16 (0.218)***** |
| *Spectral heterogeneity* | | |
| MNDWI_April_ | **-0.53 (0.053)***** | **1.12 (0.111)***** |
| *Medians* | | |
| Green_April_ | **8.12 (1.113)***** | **8.01 (1.114)***** |
| NIR_July_ | **-0.88 (0.357)*** | **-0.88 (0.357)*** |
| NDVI_April_ | **0.66 (0.153)***** | **0.65 (0.153)***** |
| MNDWI_April_ | **-1.89 (0.305)***** | **-1.62 (0.305)***** |
| *Sampling effort* | | |
| Number of visits | **0.0004 (0.00005)***** | **0.0004 (0.00005)***** |

**Table A7.** Coefficients, their standard errors (in the parentheses), and significance (asterisks) of individual model terms of the final GLMs including interactions with landscape types. Predictors computed using unclassified Landsat 8 predictors, from 30 m resolution rasters, and on large squares. Significant values are bold, with the significance level coded as follows: < 0.001 ‘***’, < 0.01 ‘**’, < 0.05 ‘*’.

| **Unclassified predictors, large squares, 30 m resolution, interactions with landscape** | | |
| --- | --- | --- |
| **Model term** | **Model with CV** | **Model with StDev** |
| Dev. explained, AIC | 58%, 1729.0 | 59%, 1732.4 |
| Intercept | **3.49 (0.304)***** | **3.98 (0.328)***** |
| *Spectral heterogeneity* | | |
| Green_July_ | 0.19 (0.168) | -5.13 (3.022) |
| NIR_April_ | -0.36 (0.219) | -1.25 (1.526) |
| NDVI_July_ | - | 0.72 (0.393) |
| MNDWI_April_ | **-0.62 (0.068)***** | **0.91 (0.226)***** |
| *Medians* | | |
| Green_April_ | **7.15 (1.374)***** | **7.05 (1.580)***** |
| NIR_July_ | - | -0.65 (0.434) |
| NDVI_April_ | **0.40 (0.171)*** | 0.45 (0.231) |
| MNDWI_April_ | **-2.28 (0.385)***** | **-1.67 (0.382)***** |
| MNDWI_July_ | **1.42 (0.475)**** | **1.31 (0.486)**** |
| *Landscape types* | | |
| LT-Coniferous | -0.37 (0.405) | -0.64 (0.468) |
| LT-Leaves | -0.43 (0.984) | 0.73 (1.436) |
| LT-Urban | **-1.22 (0.384)**** | **-1.39 (0.446)**** |
| *Interactions of spectral heterogeneity with landscape types* | | |
| Green_July_: LT-Coniferous | 0.58 (0.300) | - |
| Green_July_: LT-Leaves | -0.32 (0.468) | - |
| Green_July_: LT-Urban | **-0.65 (0.324)*** | - |
| NIR_April_: LT-Coniferous | - | 2.69 (2.088) |
| NIR_April_: LT-Leaves | - | -6.07 (4.670) |
| NIR_April_: LT-Urban | - | **5.57 (2.599)*** |
| NDVI_July_: LT-Coniferous | - | 0.21 (0.603) |
| NDVI_July_: LT-Leaves | - | -1.22 (1.171) |
| NDVI_July_: LT-Urban | - | **-1.81 (0.806)*** |
| *Interactions of medians with landscape types* | | |
| MNDWI_July_: LT-Coniferous | -0.36 (0.823) | -0.91 (0.859) |
| MNDWI_July_: LT-Leaves | -1.11 (1.776) | 0.13 (2.179) |
| MNDWI_July_: LT-Urban | **-3.00 (0.872)***** | **-2.69 (0.864)**** |
| *Sampling effort* | | |
| Number of visits | **0.0004 (0.00006)***** | **0.0005 (0.00006)***** |

**Table A8.** Coefficients, their standard errors (in the parentheses), and significance (asterisks) of individual model terms of the final GLMs using unclassified Landsat 8 predictors, computed from 100 m resolution rasters on small squares. Significant values are bold, with the significance level coded as follows: < 0.001 ‘***’, < 0.01 ‘**’, < 0.05 ‘*’.

| **Unclassified predictors, small squares, 100 m resolution** | | | |
| --- | --- | --- | --- |
| **Model term** | **Model with Rao** | **Model with CV** | **Model with StDev** |
| Dev. explained, AIC | 17%, 3571.8 | 22%, 3538.7 | 22%, 3541.7 |
| Intercept | **2.78 (0.208)***** | **1.61 (0.304)***** | **1.76 (0.289)***** |
| *Spectral heterogeneity* | | | |
| Green_April_ | - | - | 3.97 (2.683) |
| Green_July_ | - | **0.68 (0.161)***** | - |
| NIR_April_ | **3.06 (0.904)***** | - | - |
| NDVI_July_ | - | **-0.45 (0.212)*** | **0.96 (0.437)*** |
| MNDWI_April_ | - | **-0.72 (0.111)***** | **0.94 (0.249)***** |
| *Medians* | | | |
| Green_April_ | - | **7.82 (1.686)***** | **4.65 (1.764)**** |
| NIR_April_ | **2.35 (0.793)**** | - | - |
| NIR_July_ | **-1.75 (0.854)*** | - | - |
| NDVI_April_ | **-0.83 (0.337)*** | **0.73 (0.211)***** | **0.56 (0.211)**** |
| NDVI_July_ | **1.03 (0.310)***** | - | **0.62 (0.206)**** |
| MNDWI_April_ | **-1.37 (0.52)**** | **-2.32 (0.429)***** | **-1.61 (0.478)***** |
| *Sampling effort* | | | |
| Number of visits | **0.0073 (0.00102)***** | **0.0075 (0.00098)***** | **0.0073 (0.00098)***** |

**Table A9.** Coefficients, their standard errors (in the parentheses), and significance (asterisks) of individual model terms of the final GLMs including interactions with landscape types. Predictors computed using unclassified Landsat 8 predictors, from 100 m resolution rasters, and on small squares. Significant values are bold, with the significance level coded as follows: < 0.001 ‘***’, < 0.01 ‘**’, < 0.05 ‘*’.

| **Unclassified predictors, small squares, 100 m resolution, interactions with landscape** | | | |
| --- | --- | --- | --- |
| **Model term** | **Model with Rao** | **Model with CV** | **Model with StDev** |
| Dev. explained, AIC | 24%, 3560.0 | 26%, 3537.9 | 27%, 3539.1 |
| Intercept | **4.48 (0.373)***** | **2.49 (0.576)***** | **3.27 (0.476)***** |
| *Spectral heterogeneity* | | | |
| Green_April_ | **-6.2 (3.12)*** | - | - |
| Green_July_ | -2.01 (4.649) | 0.15 (0.338) | 3.17 (4.657) |
| NIR_April_ | **2.78 (1.073)**** | **-0.79 (0.33)*** | -2.81 (1.494) |
| MNDWI_April_ | - | **-0.74 (0.121)***** | **1.44 (0.265)***** |
| *Medians* | | | |
| Green_April_ | - | **9.48 (4.254)*** | 4.59 (2.765) |
| NIR_April_ | 0.63 (0.943) | **-2.37 (1.021)*** | 0.08 (0.707) |
| NIR_July_ | -0.37 (1.075) | - | -1.21 (0.834) |
| NDVI_April_ | **-1.27 (0.377)***** | 0.99 (0.509) | - |
| NDVI_July_ | 0.24 (0.39) | - | 0.39 (0.323) |
| MNDWI_April_ | - | **-1.86 (0.533)***** | -1 (0.561) |
| *Landscape types* | | | |
| LT-Coniferous | -1.48 (1.396) | -0.71 (0.45) | -2.38 (1.349) |
| LT-Leaves | -0.2 (1.682) | -0.49 (0.514) | -0.03 (1.608) |
| LT-Urban | **-2.38 (0.499)***** | **-1.06 (0.361)**** | **-1.82 (0.469)***** |
| *Interactions of spectral heterogeneity with landscape types* | | | |
| Green_July_: LT-Coniferous | **24.83 (10.278)*** | **1.28 (0.478)**** | **31.32 (10.42)**** |
| Green_July_: LT-Leaves | 13.18 (10.988) | 0.64 (0.482) | 10.61 (9.732) |
| Green_July_: LT-Urban | **20.77 (6.687)**** | 0.61 (0.437) | 6.66 (6.447) |
| *Interactions of medians with landscape types* | | | |
| NIR_April_: LT-Coniferous | 2.18 (2.538) | 1 (1.738) | 0.15 (1.894) |
| NIR_April_: LT-Leaves | -0.79 (3.614) | 1.1 (2.194) | -0.17 (2.875) |
| NIR_April_: LT-Urban | **6.75 (1.337)***** | **2.93 (1.103)**** | **3.63 (1.062)***** |
| NIR_July_: LT-Coniferous | -3.44 (2.097) | - | - |
| NIR_July_: LT-Leaves | -0.61 (2.889) | - | - |
| NIR_July_: LT-Urban | **-4.84 (1.895)*** | - | - |
| NDVI_July_: LT-Coniferous | 1.97 (1.6) | - | 2.2 (1.4) |
| NDVI_July_: LT-Leaves | 0.39 (1.645) | - | -0.16 (1.387) |
| NDVI_July_: LT-Urban | **2.49 (0.528)***** | **-** | **1.02 (0.382)**** |
| *Sampling effort* |  |  |  |
| Number of visits | **0.0078 (0.00099)***** | **0.0072 (0.00097)***** | **0.0075 (0.00096)***** |

**Table A10.** Coefficients, their standard errors (in the parentheses), and significance (asterisks) of individual model terms of the final GLMs using unclassified Landsat 8 predictors, computed from 30 m resolution rasters on small squares. Significant values are bold, with the significance level coded as follows: < 0.001 ‘***’, < 0.01 ‘**’, < 0.05 ‘*’.

| **Unclassified predictors, small squares, 30 m resolution** | | |
| --- | --- | --- |
| **Model term** | **Model with CV** | **Model with StDev** |
| Dev. explained, AIC | 23%, 3538.7 | 22%, 3543.4 |
| Intercept | 1.92 (0.293)*** | 1.57 (0.311)*** |
| *Spectral heterogeneity* | | |
| Green_April_ | - | 4.82 (2.779) |
| Green_July_ | 0.59 (0.155)*** | - |
| NIR_April_ | - | -1.96 (1.367) |
| NDVI_April_ | -0.49 (0.299) | - |
| NDVI_July_ | - | 1.12 (0.448)* |
| MNDWI_April_ | -0.74 (0.145)*** | 1.05 (0.278)*** |
| *Medians* | | |
| Green_April_ | **4.84 (2.154)*** | **5.25 (1.803)**** |
| NIR_April_ | **1.15 (0.513)*** | - |
| NDVI_April_ | - | **0.84 (0.265)**** |
| NDVI_July_ | **0.52 (0.209)*** | **0.59 (0.199)**** |
| MNDWI_April_ | **-1.71 (0.486)***** | **-1.76 (0.495)***** |
| *Sampling effort* | | |
| Number of visits | **0.0076 (0.00098)***** | **0.0072 (0.00098)***** |

**Table A11.** Coefficients, their standard errors (in the parentheses), and significance (asterisks) of individual model terms of the final GLMs including interactions with landscape types. Predictors computed using unclassified Landsat 8 predictors, from 30 m resolution rasters, and on small squares. Significant values are bold, with the significance level coded as follows: < 0.001 ‘***’, < 0.01 ‘**’, < 0.05 ‘*’.

| **Unclassified predictors, small squares, 30 m resolution, interactions with landscape** | | |
| --- | --- | --- |
| **Model term** | **Model with CV** | **Model with StDev** |
| Dev. explained, AIC | 24%, 3535.6 | 28%, 3543.5 |
| Intercept | **2.39 (0.295)***** | **2.49 (0.478)***** |
| *Spectral heterogeneity* | | |
| Green_April_ | - | -1.66 (3.697) |
| Green_July_ | **0.63 (0.152)***** | - |
| NDVI_April_ | **-0.75 (0.246)**** | - |
| NDVI_July_ | - | **1.35 (0.538)*** |
| MNDWI_April_ | **-0.78 (0.133)***** | **0.66 (0.255)**** |
| *Medians* | | |
| Green_April_ | **7.84 (1.959)***** | **6.68 (2.710)*** |
| NIR_April_ | - | -0.69 (0.695) |
| NIR_July_ | - | 0.22 (1.046) |
| NDVI_July_ | - | 0.27 (0.349) |
| MNDWI_April_ | **-1.84 (0.490)***** | **-1.44 (0.595)*** |
| *Landscape types* | | |
| LT-Coniferous | 0.03 (0.056) | **-2.95 (1.395)*** |
| LT-Leaves | -0.01 (0.053) | 0.49 (1.684) |
| LT-Urban | **-0.14 (0.040)***** | **-1.29 (0.472)**** |
| *Interactions of spectral heterogeneity with landscape types* | | |
| Green_April_: LT-Coniferous | - | **20.82 (7.995)**** |
| Green_April_: LT-Leaves | - | 5.74 (10.950) |
| Green_April_: LT-Urban | - | 5.67 (6.207) |
| *Interactions of medians with landscape types* | | |
| NIR_April_: LT-Coniferous | - | 3.37 (2.539) |
| NIR_April_: LT-Leaves | - | -1.38 (3.587) |
| NIR_April_: LT-Urban | - | **4.6 (1.278)***** |
| NIR_July_: LT-Coniferous | - | **-4.78 (2.071)*** |
| NIR_July_: LT-Leaves | - | -0.47 (2.850) |
| NIR_July_: LT-Urban | - | -3.21 (1.788) |
| NDVI_July_: LT-Coniferous | - | **4.06 (1.633)*** |
| NDVI_July_: LT-Leaves | - | -0.20 (1.645) |
| NDVI_July_: LT-Urban | - | **1.43 (0.510)**** |
| *Sampling effort* | | |
| Number of visits | **0.0072 (0.00097)***** | **0.0075 (0.00096)***** |


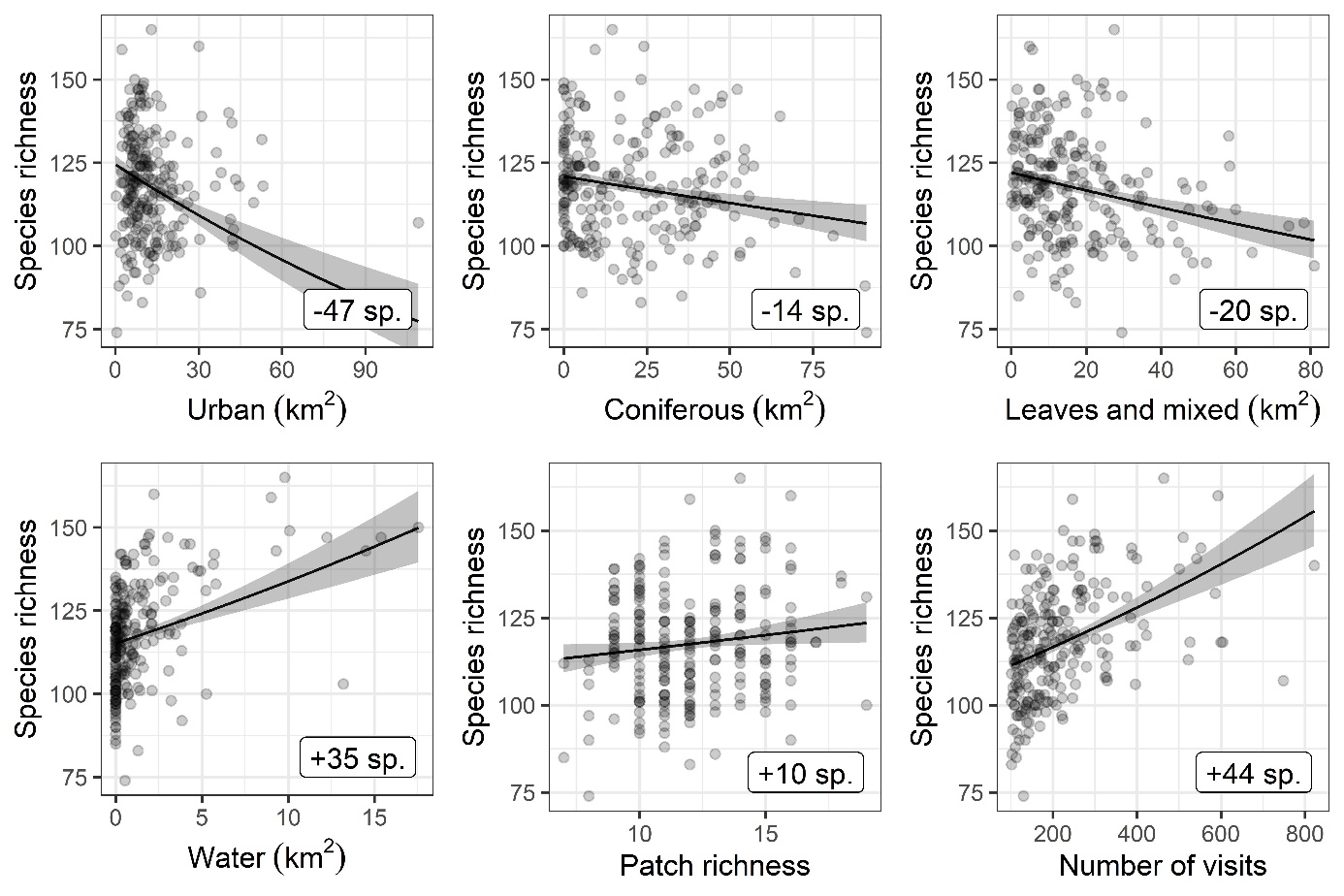


**Fig A9.** Predictor effect plots for the GLM of bird species richness on classified Corine predictors, based on large squares. Each predictor is evaluated while keeping all other predictors at their average observed value. The values in the lower right corners represent the difference between the mean predicted species richness at the maximum and minimum observed value of the predictor.


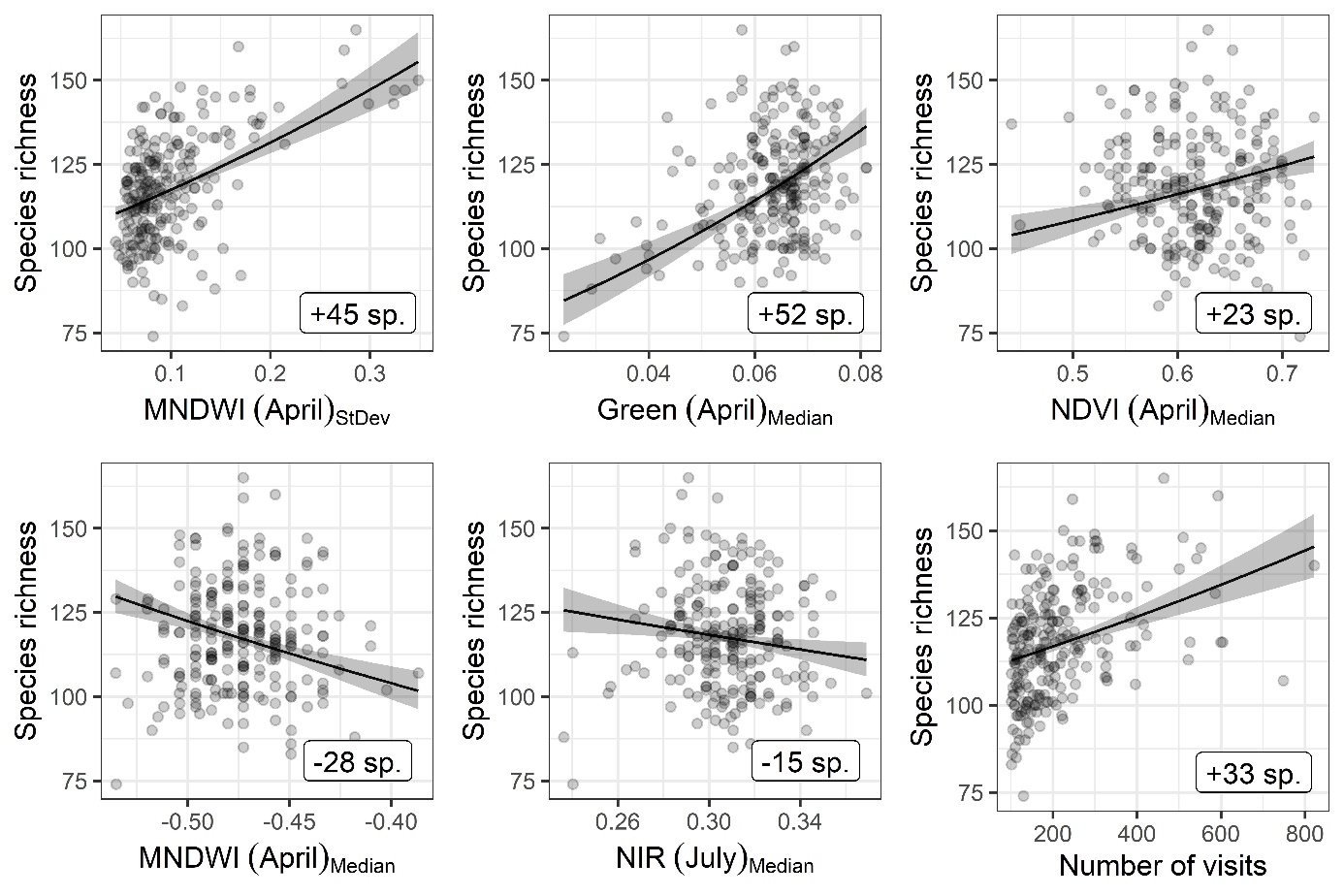


**Fig A10.** Predictor effect plots for the GLM of bird species richness on unclassified Landsat 8 predictors, based on large squares. Each predictor is evaluated while keeping all other predictors at their average observed value.


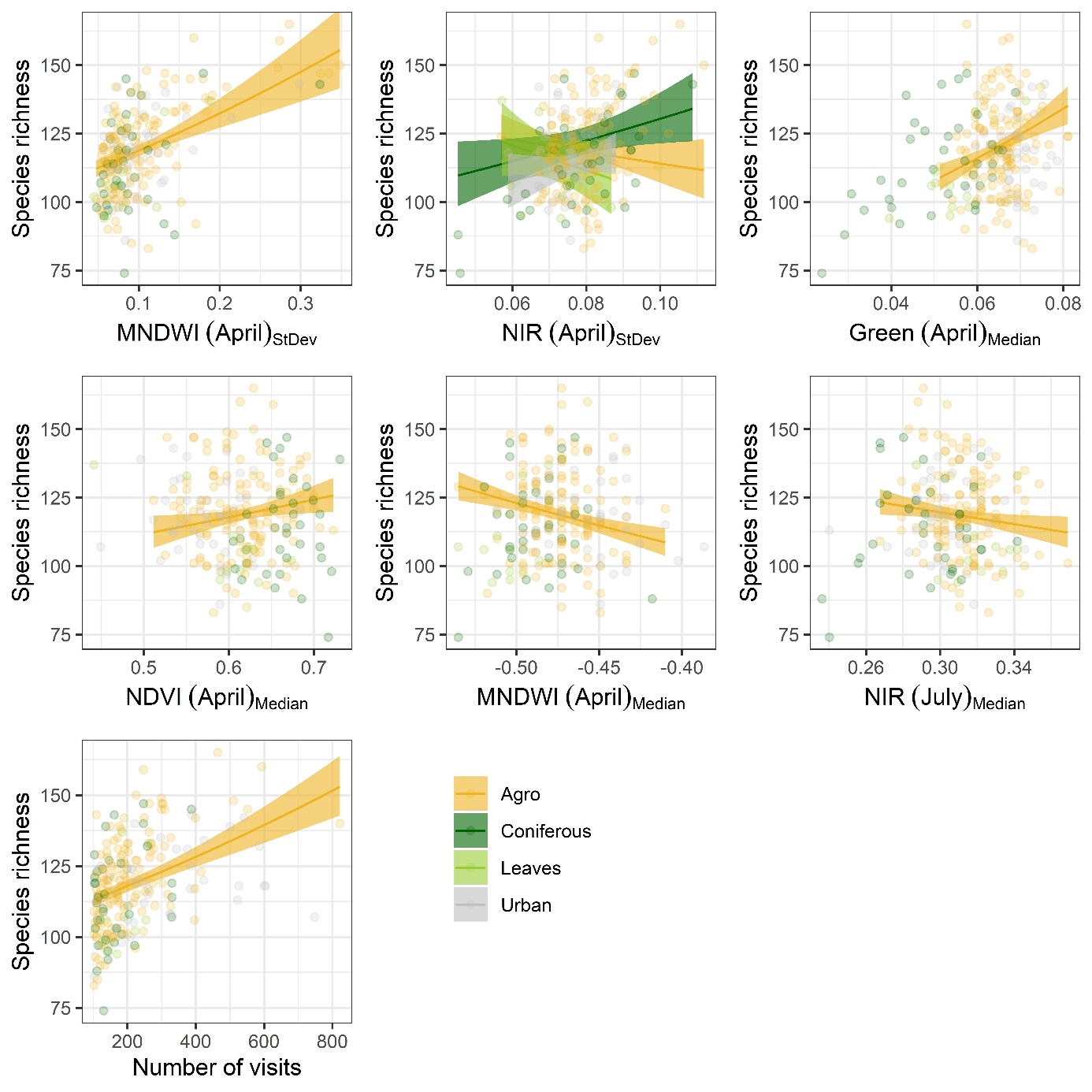


**Fig A11.** Predictor effect plots for the GLM of bird species richness on unclassified Landsat 8 predictors interacting with landscape types, based on large squares. Each predictor is evaluated while keeping all other predictors at their average observed value in a given landscape type category. For predictors without significant interaction with landscape type, the landscape type was fixed to “Agro”.
